# MED13-dependent signaling from the heart confers leanness by enhancing metabolism in adipose tissue and liver

**DOI:** 10.15252/emmm.201404218

**Published:** 2014-11-24

**Authors:** Kedryn K Baskin, Chad E Grueter, Christine M Kusminski, William L Holland, Angie L Bookout, Santosh Satapati, Y Megan Kong, Shawn C Burgess, Craig R Malloy, Philipp E Scherer, Christopher B Newgard, Rhonda Bassel-Duby, Eric N Olson

**Affiliations:** 1 Department of Molecular Biology, University of Texas Southwestern Medical CenterDallas, TX, USA; 2 Division of Cardiovascular Medicine, Department of Internal Medicine, University of Iowa Carver College of MedicineIowa City, IA, USA; 3 Touchstone Diabetes Center, Department of Internal Medicine, University of Texas Southwestern Medical CenterDallas, TX, USA; 4 Advanced Imaging Research Center, University of Texas Southwestern Medical CenterDallas, TX, USA; 5 Department of Pharmacology, University of Texas Southwestern Medical CenterDallas, TX, USA; 6 Department of Radiology, University of Texas Southwestern Medical CenterDallas, TX, USA; 7 Department of Molecular Biophysics, University of Texas Southwestern Medical CenterDallas, TX, USA; 8 Department of Cell Biology, University of Texas Southwestern Medical CenterDallas, TX, USA; 9 Sarah W. Stedman Nutrition and Metabolism Center, Duke UniversityDurham, NC, USA; 10 Duke Molecular Physiology Institute, Duke UniversityDurham, NC, USA; 11 Department of Pharmacology and Cancer Biology, Department of Medicine, Duke UniversityDurham, NC, USA; 12 Hamon Center for Regenerative Science and MedicineDallas, TX, USA

**Keywords:** energy homeostasis, mediator complex, metabolic flexibility, metabolic gene expression, metabolism

## Abstract

The heart requires a continuous supply of energy but has little capacity for energy storage and thus relies on exogenous metabolic sources. We previously showed that cardiac MED13 modulates systemic energy homeostasis in mice. Here, we sought to define the extra-cardiac tissue(s) that respond to cardiac MED13 signaling. We show that cardiac overexpression of MED13 in transgenic (MED13cTg) mice confers a lean phenotype that is associated with increased lipid uptake, beta-oxidation and mitochondrial content in white adipose tissue (WAT) and liver. Cardiac expression of MED13 decreases metabolic gene expression in the heart but enhances them in WAT. Although exhibiting increased energy expenditure in the fed state, MED13cTg mice metabolically adapt to fasting. Furthermore, MED13cTg hearts oxidize fuel that is readily available, rendering them more efficient in the fed state. Parabiosis experiments in which circulations of wild-type and MED13cTg mice are joined, reveal that circulating factor(s) in MED13cTg mice promote enhanced metabolism and leanness. These findings demonstrate that MED13 acts within the heart to promote systemic energy expenditure in extra-cardiac energy depots and point to an unexplored metabolic communication system between the heart and other tissues.

See also: **M Nakamura & J Sadoshima** (December 2014)

## Introduction

Obesity has reached epidemic proportions worldwide and is associated with increased risk of cardiovascular disease, hypertension, and diabetes, resulting in enhanced morbidity and mortality (Van Gaal *et al*, 2006). Obesity is associated with a variety of abnormalities in metabolic homeostasis, including insulin resistance, glucose intolerance, hyperlipidemia, and a condition described as “metabolic inflexibility”, referring to impairment in normal switching from fatty acid to glucose utilization in the fasted to fed transition (Storlien *et al*, 2004; Harmancey *et al*, 2008).

The ability of the body to adapt to metabolic changes in both the fed and fasted states requires inter-organ communication. While it is well known that adipose tissue communicates in an endocrine manner with most other organs (Sun *et al*, 2011; Rosen & Spiegelman, 2014), it is also becoming clear that such signaling is reciprocal. The heart is particularly interesting with respect to inter-organ metabolic signaling because it requires a continual supply of energy to sustain contraction and metabolism, but stores only enough energy for a few heart beats. Under normal conditions, the heart relies primarily on oxidation of fatty acids delivered via the circulation for energy and responds to metabolic signals from adipose tissue (Karmazyn *et al*, 2008). However, under conditions of stress, and in the postprandial state, the heart shifts toward glucose metabolism. Abnormalities in cardiac energy homeostasis, as occur in diabetes and other disorders, correlate with increased incidence of contractile dysfunction and heart failure (Madrazo & Kelly, 2008).

Recently, we discovered a key role of the Mediator component MED13 in the control of systemic energy homeostasis from the heart (Grueter *et al*, 2012). The Mediator complex regulates transcription by bridging the general transcriptional machinery with specific transcription factors. This complex consists of ˜30 proteins, which exist in two major conformations that differ by the presence or absence of the CDK8 submodule. The CDK8 submodule consists of four proteins, CDK8, cyclin C, MED12, and MED13 (Conaway & Conaway, 2011). This submodule functions to inhibit transcription when bound to the core Mediator complex, and by tethering chromatin modifying proteins and transcriptional elongation factors to sites of transcription initiation (Grueter, 2013). MED13 is especially important in regulating transcription, as it serves as the molecular bridge between the core Mediator complex and the kinase submodule (Knuesel *et al*, 2009).

Our initial studies revealed an unexpected influence of the heart on systemic metabolism (Grueter *et al*, 2012). Elevated expression of MED13 within hearts of transgenic mice confers a lean phenotype with a pronounced reduction in adipose tissue mass. Conversely, knockdown of *Med13* in *Drosophila* or cardiac deletion of *Med13* in mice enhances susceptibility to diet-induced obesity (Pospisilik *et al*, 2010; Grueter *et al*, 2012; Lee *et al*, 2014). These findings raise interesting and important questions regarding the mechanism for cardiac control of systemic energy homeostasis and the tissues responsible for altered energy expenditure in response to cardiac MED13 expression. In the present study, we identify white adipose tissue (WAT) and liver as physiological targets for MED13-dependent regulation of energy homeostasis by the heart. Elevated expression of MED13 in the heart enhances metabolic gene expression, mitochondria number and energy consumption in WAT, and results in changes in metabolite profile and energy consumption in liver. MED13 also decreases cardiac metabolic gene expression and alters metabolomic profile in the heart and liver. Although energy expenditure is increased in the fed state, transgenic mice with cardiac overexpression of MED13 (MED13cTg mice) are metabolically flexible and able to adapt to fasting. Finally, using heterotypic parabiosis, we demonstrate that circulating factors in MED13cTg mice regulate WAT and liver metabolism, and wild-type (WT) mice subjected to the lean systemic milieu of MED13cTg mice acquire an enhanced metabolic phenotype. These results provide evidence that crosstalk between the heart and energy depots occurs *in vivo* and suggest that this is regulated in the heart at the level of transcription by MED13.

## Results

### Cardiac overexpression of MED13 enhances lipid metabolism in white adipose tissue

Transgenic mice with enhanced cardiac MED13 expression (MED13cTg mice) display a lean phenotype, have a 15% reduction in fat mass at 12 weeks of age on normal chow compared to WT mice, and are resistant to diet-induced obesity. Oxygen consumption and carbon dioxide production, measures of energy expenditure, were significantly increased in MED13cTg mice, primarily in the dark cycle or fed state, with no change in the respiratory exchange ratio, food intake or physical activity compared to WT littermates (Grueter *et al*, 2012). Because of the profound effects of cardiac MED13 expression on whole-body metabolism, we sought to identify the tissue(s) responsible for the enhanced metabolic state of these animals.

**Figure 1:**
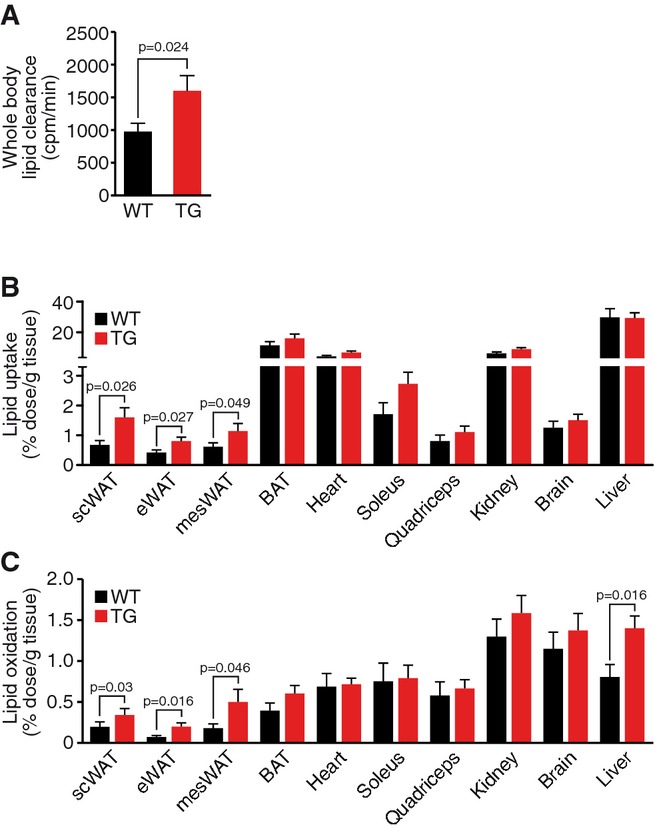
Cardiac overexpression of MED13 increases lipid metabolism in white adipose tissue Increased whole-body [^3^H]-triolein clearance in MED13cTg mice after a 3-h fast.Increased lipid ([^3^H]-triolein) uptake in MED13cTg white adipose tissue (WAT) after a 3-h fast.Increased lipid ([^3^H]-triolein) oxidation in MED13cTg WAT and liver after a 3-h fast. Data information: Data are mean ± SEM, *t*-test, *n* = 8–12.

We first analyzed lipid uptake *in vivo* using [^3^H]-triolein tracer studies. MED13cTg mice displayed a 60% increase in lipid clearance rate compared to WT littermates, measured by decreased [^3^H]-triolein in MED13cTg blood (Fig1A). In order to identify the tissue(s) responsible for the enhanced lipid clearance, we analyzed [^3^H]-triolein levels in multiple tissues. Lipid uptake in muscle and most other organs was comparable in WT and MED13cTg mice. In contrast, lipid uptake was increased in subcutaneous (scWAT), epididymal (eWAT), and mesenteric (mesWAT) white adipose tissue (WAT) of MED13cTg mice (Fig1B). In the same [^3^H]-triolein tracer studies, we also analyzed steady-state lipid oxidation and observed a significant increase in lipid oxidation in all WAT depots and in liver of MED13cTg mice (Fig1C). These experiments point to the WAT and liver as the main non-cardiac tissues responsible for the enhanced metabolic rate we observe in the MED13cTg mice.

### Regulation of WAT gene expression by cardiac MED13

**Figure 2:**
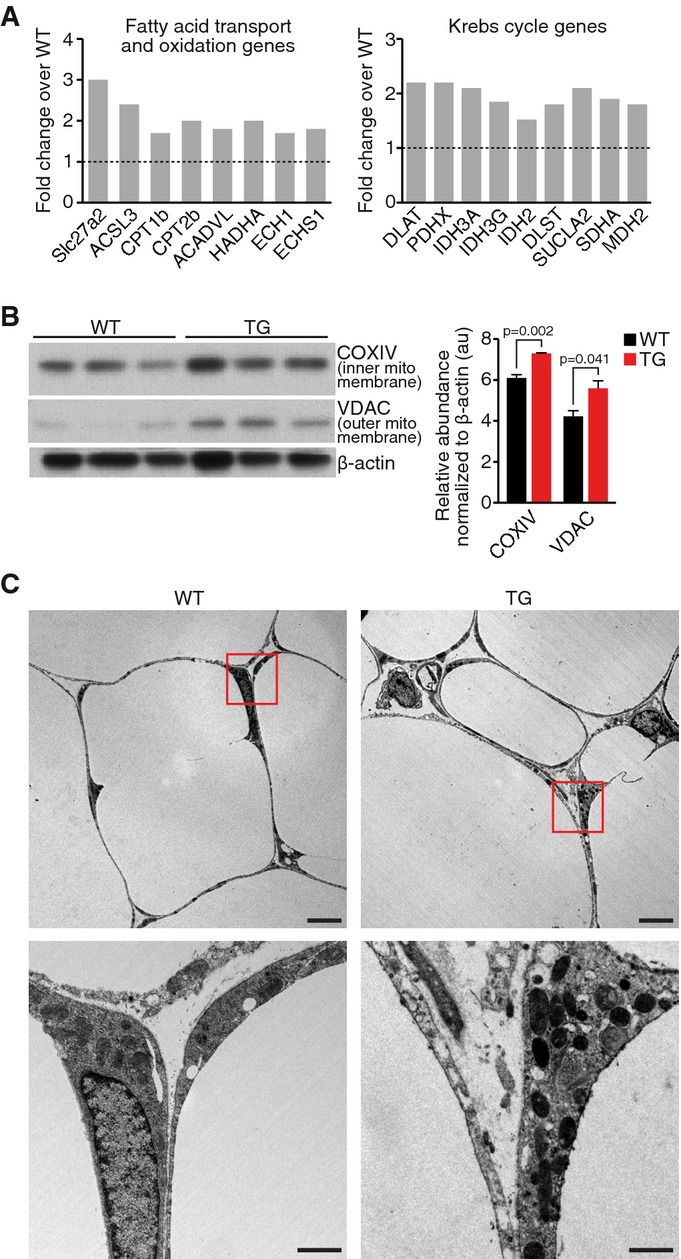
Cardiac overexpression of MED13 increases metabolic gene expression and mitochondria content in white adipose tissue Enhanced expression of genes involved in fatty acid uptake and oxidation and Krebs cycle in MED13cTg WAT determined by RNA deep sequencing, *n* = 3.Increased mitochondrial protein expression detected by Western blot analysis in MED13cTg WAT, *n* = 3. Data are mean ± SEM, *t*-test.Mitochondrial content demonstrated by transmission electron microscopy in MED13cTg WAT. Top scale bars, 5 μm; bottom scale bars, 1 μm. Source data are available online for this figure.

Studies summarized in Fig1 demonstrate that cardiac expression of MED13 selectively enhances lipid uptake and metabolism in WAT and liver, whereas other tissues are insensitive to this signaling mechanism. To further explore the mechanistic basis of the lean phenotype conferred by cardiac MED13 overexpression, we compared gene expression profiles of WAT from WT and MED13cTg mice by RNA deep sequencing. Detailed analysis of the gene expression profiles revealed over 800 significantly upregulated genes in WAT of MED13cTg mice on normal chow. Strikingly, we discovered concerted upregulation of multiple genes in the fatty acid β-oxidation pathway and in the Krebs cycle in MED13cTg WAT compared to WAT from WT littermates (Fig2A and Supplementary Fig S1). These experiments further confirm the triolein tracer experiments that revealed an increase in β-oxidation in MED13cTg WAT.

Increased mitochondrial number can enhance energy expenditure. Indeed, transmission electron microscopy revealed that MED13cTg adipocytes had increased mitochondrial content compared to WT adipocytes (Fig2C). Markers of the inner and outer mitochondrial membrane, cytochrome c oxidase or complex IV (COXIV) and voltage-dependent anion channel (VDAC), respectively, were also increased in MED13cTg WAT as determined by Western blot (Fig2B). These results indicate that overexpression of MED13 in the heart leads to increased mitochondrial content, and increased expression of oxidative enzymes in adipose tissue, resulting in an increased rate of fatty acid oxidation.

### Overexpression of cardiac MED13 augments fatty acid metabolism in liver

**Figure 3:**
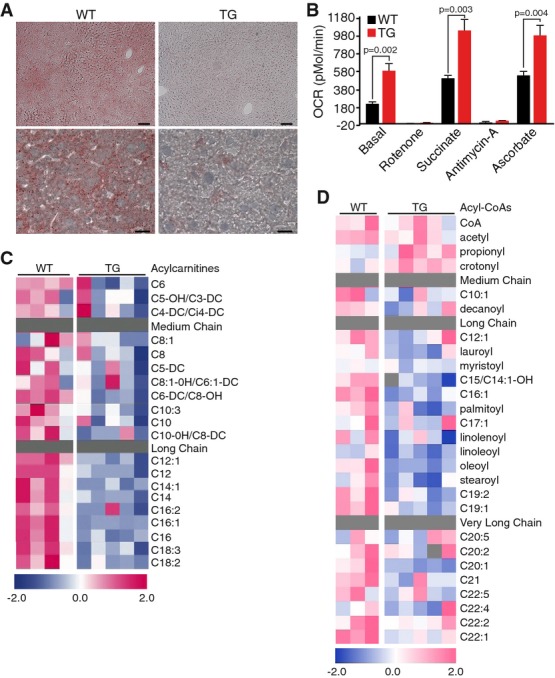
Overexpression of cardiac MED13 alters liver fatty acid metabolism ALiver from fed MED13cTg mice on normal chow accumulate less lipids than WT, indicated by decreased oil red O staining. Top scale bars, 100 μm; bottom scale bars, 20 μm.BMED13cTg liver mitochondria have higher basal oxygen consumption rates, measured using the XF24 Extracellular Flux Analyzer. Data are mean ± SEM, *t*-test, *n* = 5.C–DMED13cTg liver mitochondria have (C) decreased steady-state levels of short, medium, and long-chain acylcarnitine, and (D) decreased levels of Acyl-CoA species, measured by metabolomics.

Although MED13cTg mice display enhanced hepatic lipid oxidation (Fig1C), lipid uptake by the liver was comparable in MED13cTg and WT littermates (Fig1B). Consequently, hepatic lipid accumulation was markedly decreased in MED13cTg mice, visualized by reduced oil red O staining of intracellular lipids (Fig3A). In order to functionally assess changes in liver metabolism, we analyzed mitochondrial metabolism and function of the electron transport chain in isolated hepatic mitochondria. Under basal conditions, in the presence of substrates pyruvate and malate, hepatic mitochondria from MED13cTg mice displayed dramatically higher oxygen consumption rates (OCR) (Fig3B). Rotenone, an inhibitor of complex I of the electron transport chain, decreased OCR similarly in both WT and MED13cTg liver mitochondria. Interestingly, MED13cTg hepatic mitochondria exhibited a higher OCR in response to the complex II substrate succinate, compared to WT hepatic mitochondria. Finally, while the complex III inhibitor antimycin A repressed OCR in WT and MED13cTg mitochondria, significant increases in OCRs were observed in MED13cTg mitochondria in response to the complex IV substrate, ascorbate (Fig3B). These experiments demonstrate that MED13cTg hepatic mitochondria harbor fully intact and functional electron transport chains, and moreover, exhibit a higher mitochondrial respiratory capacity in response to a variety of substrates.

To gain further insight into metabolic changes caused by cardiac MED13 overexpression, we measured a large panel of acylcarnitines, acyl-CoAs, amino acids, organic acids (Krebs cycle intermediates), and ceramides by targeted MS/MS and GC/MS in liver from MED13cTg and WT mice, in the fed and fasted states. Tissue analyses revealed a very clear difference between MED13cTg and WT animals that is specific to the fed condition. We observed a pattern of decreased acylcarnitines in the liver of fed MED13cTg mice (Fig3C). Acyl-CoAs also tended to decrease in the fed state in MED13cTg compared to WT livers (Fig3D). These changes in the fed state occurred independent of alterations in Krebs cycle intermediates, suggesting that substrate influx to the Krebs cycle remains adequate to maintain normal levels of all intermediates (Supplementary Fig S2A). Collectively, these data indicate that overexpression of MED13 in the heart enhances hepatic lipid metabolism, particularly in the fed condition.

### Overexpression of cardiac MED13 increases metabolic fuel efficiency in the heart

**Figure 4:**
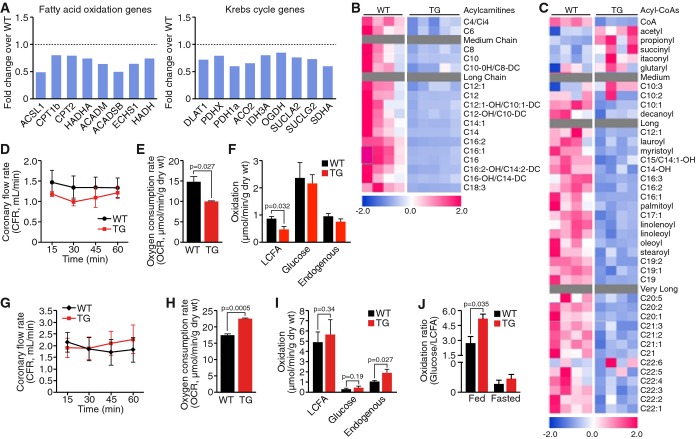
Overexpression of cardiac MED13 alters cardiac metabolism AExpression of fatty acid oxidation and Krebs cycle genes in MED13cTg heart determined by RNA deep sequencing, *n* = 3.B–CMED13cTg hearts have decreased (B) acylcarnitines and (C) long-chain acyl-CoA species in the fed state, as measured by metabolomics, *n* = 5.D–FIn conditions simulating the fed state, MED13cTg hearts displayed (D) normal coronary flow rates, (E) decreased oxygen consumption, and (F) decreased long-chain fatty acid (LCFA) substrate oxidation rates, as determined by Langendorff heart perfusions and ^13^C NMR analysis, *n* = 4–6.G–IIn conditions simulating the fasted state, MED13cTg hearts displayed (G) normal coronary flow rates, (H) increased oxygen consumption, and (I) increased endogenous substrate oxidation, as determined by Langendorff heart perfusions and ^13^C NMR analysis.JIn conditions simulating the fed state, when glucose is readily available, the oxidation ratio shifts towards a preference for glucose in MED13cTg hearts. In conditions simulating the fasted state, when LCFAs are more readily available, oxidation ratios are similar in WT and MED13cTg hearts, *n* = 4–6. Data information: Data are mean ± SEM, *t*-test.

Given that MED13 is a transcriptional regulator, we investigated cardiac gene expression in detail, by performing RNA deep sequencing on ventricles isolated from MED13cTg mice. We discovered that many genes in the fatty acid β-oxidation pathway and in the Krebs cycle were downregulated in MED13cTg hearts compared to hearts from WT littermates (Fig4A and Supplementary Fig S3A and B).

To determine the functional consequence of decreased metabolic gene expression, we investigated cardiac metabolism using Langendorff heart perfusions to quantify substrate utilization in the heart. We first verified that the exogenous substrates provided to the heart in perfusion experiments were within the physiological range detected in WT and MED13cTg mouse serum. The concentrations of glucose, free fatty acids, triglycerides, and ketones were the same in WT and MED13cTg serum in the postprandial state (Supplementary Fig S3D). We perfused hearts under conditions that simulated the fed state with Krebs buffer supplemented with [1,6-^13^C] 10 mM glucose and uniformly labeled [U-^13^C] 0.1 mM long-chain fatty acids (LCFA) in the presence of insulin. Throughout the 60-min perfusion experiments, we did not detect differences in coronary flow rates (Fig4D), but oxygen consumption rates (OCR) were decreased in MED13cTg hearts (Fig4E), suggesting that substrate utilization is either decreased or altered in MED13cTg hearts in the fed state.

Substrate oxidation was determined by ^13^C-NMR analysis of perfused hearts; a representative ^13^C-NMR spectra is provided in Supplementary Fig S3E. In the fed state, oxidation of exogenous uniformly labeled [U-^13^C] long-chain fatty acids was decreased in MED13cTg hearts, while oxidation of exogenous [1,6-^13^C] glucose and endogenous substrate(s) was similar in WT and MED13cTg hearts (Fig4F). Thus, when perfused under the fed-like state in the presence of insulin, a higher concentration of glucose and lower concentration of LCFA, MED13cTg hearts consume less oxygen than WT hearts as a result of oxidizing less fatty acids. These results suggest that hearts from MED13cTg mice prefer to oxidize substrate that is more readily available.

In a separate set of experiments, we perfused hearts under conditions that simulate the fasted state with Krebs buffer supplemented with [1,6-^13^C] 8 mM glucose and uniformly labeled [U-^13^C] 0.4 mM long-chain fatty acids. As in the fed state, we did not detect differences in coronary flow rates (Fig4G), but oxygen consumption rates (OCR) were increased in MED13cTg hearts (Fig4H), indicating that substrate utilization is increased in MED13cTg hearts in the fasted state. Although we were unable to detect changes in oxidation of exogenous [1,6-^13^C] glucose and uniformly labeled [U-^13^C] long-chain fatty acids, determined by ^13^C-NMR analysis of perfused hearts, oxidation of endogenous substrate(s) was significantly increased in MED13cTg hearts in the fasted-like state (Fig4I).

**Figure 5:**
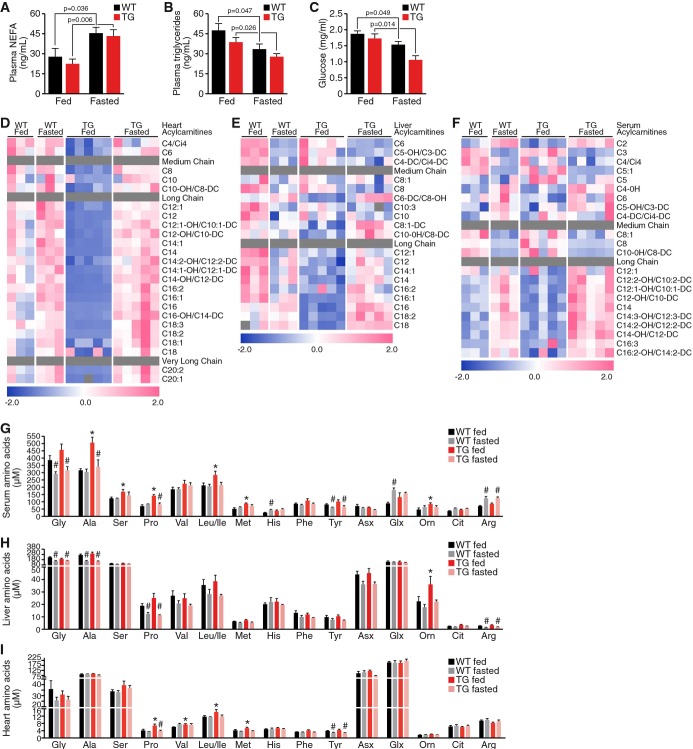
MED13cTg mice maintain the ability to adapt metabolically to fasting A–CNon-esterified free fatty acids (NEFA) (A), triglycerides (B), and glucose levels (C) in serum from fed and fasted mice.D–FAcylcarnitine species in (D) heart, (E) liver, and (F) serum from fed and fasted mice.G–IAmino acids in (G) serum, (H) liver, and (I) heart from fed and fasted mice, *n* = 5. Data information: Data are mean ± SEM, two-way ANOVA followed by Tukey's test, *P < 0.05 TG fed versus WT fed, ^#^*P* < 0.05 fed versus fasted.

To gain insight into the possible endogenous substrates utilized by MED13cTg hearts, we performed metabolomics on ventricles isolated from WT and MED13cTg mice, in the fed and fasted states. We observed dramatic decreases in a large array of acylcarnitine species in fed MED13cTg compared to fed WT hearts (Fig4B), while there was a trend of increased long-chain acylcarnitine species in fasted MED13cTg compared to fasted WT hearts (Fig5D and Supplementary Table S3). A similar but less dramatic decline was also observed in medium, long-chain, and very long-chain acyl-CoAs in hearts from fed MED13cTg compared to fed WT mice (Fig4C), whereas there was a trend of increased long-chain and very long-chain acyl-CoAs in fasted MED13cTg compared to fasted WT hearts (Supplementary Fig S4A and Supplementary Table S4). Thus, in the fed state, metabolic gene expression is decreased in MED13cTg hearts, long-chain fatty acid utilization is decreased, and there is a decrease in accumulation of intermediates of fatty acid metabolism. However, in MED13cTg hearts in the fasted state, endogenous substrate utilization is increased and there is a trend of increased accumulation of intermediates of fatty acid metabolism. These results demonstrate that MED13 plays a central role in the regulation of cardiac metabolism by increasing utilization of fuel that is most readily available with alternating nutrient states (Fig4J).

### MED13cTg mice maintain the ability to adapt metabolically to fasting

Inter-organ communication is required to orchestrate an adaptive response to metabolic stress such as starvation. Short-term fasting (< 24 h) mobilizes triglyceride stores from adipose tissue, resulting in increased circulating fatty acids. Some of the fatty acids are taken up by the liver, converted to acetyl CoA, and then either oxidized or secreted into the blood in the form of ketone bodies. Hepatic glycogenolysis supplies the brain with glucose, and muscle supplies gluconeogenic amino acids for hepatic gluconeogenesis (Cahill, 1976). Because metabolism is enhanced and triglyceride storage is diminished in MED13cTg mice, we investigated whether these mice were still able to adapt to short-term metabolic stress.

We first investigated circulating levels of non-esterified free fatty acids (NEFA), triglycerides, and glucose in MED13cTg mice after an overnight fast (about 18 h). There was no significant difference in NEFA, triglycerides, or glucose or in MED13cTg serum in the fed or fasted state (Fig5A–C). Even though MED13cTg mice display enhanced metabolic rates in the fed state, NEFA levels were still increased after fasting (Fig5A). Additionally, serum triglycerides and glucose levels decreased in fasted MED13cTg mice, to comparable levels seen in WT serum (Fig5B and C).

Remarkably, levels of medium, long-chain, and very long-chain acylcarnitine species that fell precipitously in liver and heart of fed MED13cTg mice (Fig5D and E, Supplementary Tables S1 and S3) were maintained at the same levels in the serum of fed MED13cTg and WT mice (Fig5F, Supplementary Table S5). Clear increases in circulating acylcarnitines were observed in response to fasting in both WT and MED13cTg mice, suggesting normal switching of tissues from glucose to lipid metabolism in response to dietary status in MED13cTg mice. With regard to the other analyte modules, Krebs cycle intermediates were not different in MED13cTg compared to WT mice in either the fed or fasted states, in heart or in liver (Supplementary Fig S2A and B). This suggests that despite the large decreases in lipid-derived acylcarnitine and acyl-CoA intermediates, substrate influx to the Krebs cycle remains adequate to maintain normal levels of all intermediates.

Similarly, no genotype or diet differences were noted in the levels of most ceramide species, with the following exceptions. In liver, C16, C18, C20, C26:1, and d18:1/C26 ceramides were decreased in fed MED13cTg versus fed WT mice, whereas these analytes were not different when comparing livers from fasted MED13cTg and WT mice (Supplementary Fig S2C). Interestingly, C16, C18, and C20 ceramides showed a trend to increase in hearts of fed MED13cTg versus fed WT mice, with few changes in the other ceramide species in fed or fasted mice (Supplementary Fig S2D). Amino acid profiling revealed increases in the branched chain amino acids valine, leucine, and isoleucine, and neutral amino acids, proline and methionine in heart samples from fed MED13cTg mice compared to fed WT mice; however, these metabolites were not different in hearts in the fasted state (FigI). These same amino acids were elevated in serum of fed MED13cTg compared to fed WT mice (Fig5G). Changes in these metabolites were not evident in liver in the fed or fasted states, but the urea cycle intermediate ornithine was elevated in fed MED13cTg mice compared to fed WT mice (Fig5H). Interestingly, propionyl CoA, a product of methionine, valine, and isoleucine catabolism, was elevated in heart (Supplementary Fig S4A, Supplementary Table S2) and liver (Supplementary Fig S4B, Supplementary Table S4) of fed MED13cTg mice compared to fed WT mice. Additionally, C5 acylcarnitine, also a product of BCAA catabolism, was increased in plasma of MED13cTg mice (Fig5F). These findings may suggest that amino acid mobilization and utilization contribute to maintenance of metabolic homeostasis and normal levels of Krebs cycle intermediates in MED13cTg mice.

We interpret these metabolic signatures to mean that expression of MED13 in the heart regulates not only cardiac metabolism, but also whole-body metabolic homeostasis. Normally, feeding causes lowering of circulating fatty acid levels, in part via suppression of lipolysis, and this coupled with a rise in glucose-derived malonyl CoA, a potent allosteric inhibitor of CPT1, causes fatty acid oxidation to be decreased. Our metabolic profiling shows that MED13cTg fed mice experience a significant decline in a broad array of intermediates of fatty acid oxidation (acyl-CoAs and acylcarnitines), particularly in heart. Indeed, we observed a decrease in oxygen consumption and long-chain fatty acid oxidation in hearts of MED13cTg mice in the fed state. Decreased fatty acid oxidation in the fed state in MED13cTg mice, however, does not appear to involve a surfeit of malonyl CoA, as direct measurements of malonyl CoA levels by LC-MS/MS in fed MED13cTg compared to WT hearts revealed no differences in this important CPT1 allosteric inhibitor (Supplementary Fig S3F). The decrease in heart and liver acylcarnitines and acyl-CoAs that we observed is not due to substrate depletion, as the levels of circulating FFA are not different in fed MED13cTg compared to fed WT mice. Our studies imply potential compensation of non-cardiac tissues to counter the depleted acyl-CoA and acylcarnitine pool in the heart, as suggested by maintenance of normal circulating levels of acylcarnitines. Recent studies have indicated that circulating acylcarnitines can serve as an important fuel in sustained exercise (Seiler *et al*, 2014), and their increased release by liver and heart may be a mechanism that allows sustained cardiac function under conditions of substrate pool depletion.

### Circulating factor(s) regulate enhanced WAT and liver metabolism and contribute to the lean phenotype of MED13cTg mice

**Figure 6:**
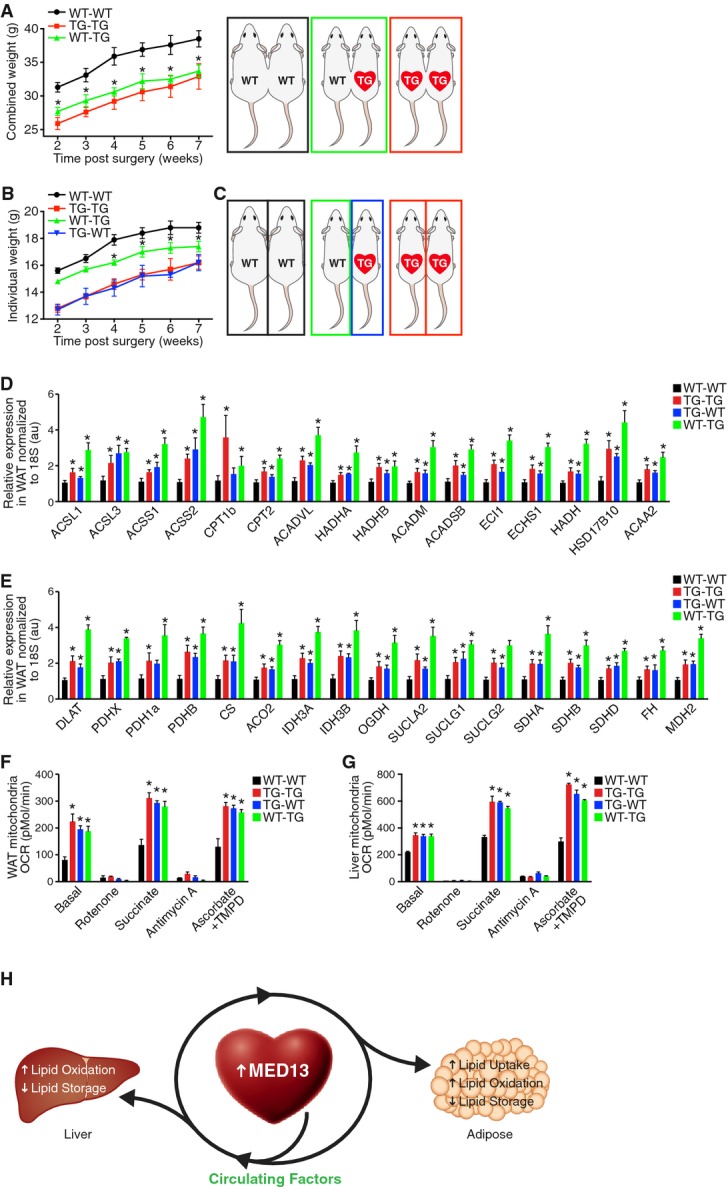
Circulating factor(s) regulate enhanced WAT and liver metabolism and contribute to the lean phenotype of MED13cTg mice AHeterotypic parabiotic pairs gain less weight than isotypic WT parabiotic pairs.BHeterotypic TG parabiots weigh the same as isotypic TG parabiots, while heterotypic WT parabiots weigh significantly less than isotypic WT parabiots.CSchematic of isotypic and heterotypic parabiosis.D,EExpression of (D) fatty acid oxidation genes and (E) Krebs cycle genes in WAT of parabiots as determined by qPCR. 18S rRNA was used for normalization.F,GOxygen consumption is significantly increased in mitochondria isolated from (F) WAT and (G) liver of isotypic TG and heterotypic TG and WT parabiots, *n* = 6 pair (12 mice) per group.HCardiac MED13 increases lipid uptake in adipose tissue, and increases lipid oxidation and decreases lipid storage in both adipose tissue and liver. Inter-organ communication in MED13cTg mice is controlled by circulating factors that enhance WAT and liver metabolism resulting in a lean phenotype. Data information: Data are mean ± SEM, two-way ANOVA followed by Dunnett's test, *P < 0.05 versus isotypic WT-WT parabiots.

To explore how metabolic homeostasis is regulated and to investigate inter-organ communication in MED13cTg mice, we tested whether circulating factors in MED13cTg mice regulate metabolism in extra-cardiac energy depots. We performed isotypic (WT-WT and TG-TG) and heterotypic (WT-TG) parabiosis experiments with WT and MED13cTg (TG) mice (Supplementary Fig S5). Male mice were surgically conjoined at 4 weeks of age, allowed 2 weeks to adapt and recover from surgery, and followed for an additional 5 weeks. All parabiotic mouse pairs gained weight over time; however, the WT-TG heterotypic parabiotic pairs weighed significantly less than the WT-WT isotypic pairs (Fig6A). Because TG mice weigh less than WT mice (Grueter *et al*, 2012), we determined individual parabiot weights throughout the course of the experiment by weighing conjoined mice on conjoined scales. While heterotypic TG parabiots (designated as TG-WT, shown in blue) weighed the same as isotypic TG parabiots (designated as TG-TG, shown in red), heterotypic WT parabiots (designated as WT-TG, shown in green) weighed significantly less than isotypic WT parabiots (designated as WT-WT, shown in black) (Fig6B, Supplementary Fig S5). These results demonstrate that WT mice acquire a lean systemic milieu when subjected to heterotypic parabiosis and suggest that circulating factor(s) in MED13cTg mice are, at least in part, responsible for the lean phenotype.

To determine how circulating factors in MED13cTg mice regulate body weight of WT mice, we investigated WAT and liver of parabiots, the tissues largely responsible for enhanced metabolic rate in MED13cTg mice. We quantified gene expression in WAT of isotypic and heterotypic parabiots and discovered that 7 weeks of conjoined circulation leads to remodeling of the genetic signatures in WAT. Expression of fatty acid oxidation genes (Fig6D) and Krebs cycle genes (Fig6E) were significantly increased in isotypic TG WAT, similar to what we observed in MED13cTg WAT by RNA-seq (Fig2). Fatty acid oxidation and Krebs cycle genes remain highly expressed in WAT of heterotypic TG parabiots and are even more highly expressed in WAT of heterotypic WT parabiots (Fig6D and E). These results indicate that circulating factors in MED13cTg mice regulate metabolic gene expression in WAT.

We then investigated the metabolic consequences of enhanced gene expression by measuring metabolic rates in WAT and liver in parabiots. We isolated mitochondria from isotypic and heterotypic parabiot WAT and liver and quantified oxygen consumption rates (OCR). Under basal conditions, in the presence of substrates pyruvate and malate, adipose and hepatic mitochondria from isotypic and heterotypic TG parabiots displayed dramatically higher oxygen consumption rates than isotypic WT parabiots (Fig6F and G), similar to what we found in MED13cTg hepatic mitochondria (Fig3B). Furthermore, adipose and hepatic mitochondria from heterotypic WT parabiots had markedly enhanced basal OCR, demonstrating that circulating factors regulate not only metabolic gene expression, but also metabolic rates in WAT and liver. Additionally, adipose and hepatic mitochondria from heterotypic WT parabiots responded to substrates and inhibitors of the electron transport chain in a similar manner as mitochondria from isotypic and heterotypic TG parabiots (Fig6F and G). These experiments demonstrate that adipose and hepatic mitochondria from all parabiots harbor fully intact and functional electron transport chains. Moreover, WAT and liver from heterotypic WT parabiots metabolically remodel when subjected to the lean systemic milieu of MED13cTg mice. Taken together, these results provide strong evidence that circulating factors in MED13cTg mice are responsible for regulating WAT and liver metabolism and ultimately a lean phenotype.

## Discussion

The results of this study demonstrate that elevation of MED13 expression in the mouse heart enhances metabolic rates and mitochondrial content of WAT and liver, resulting in a lean phenotype, with distinct changes in metabolic profile that are most pronounced in the fed state. These findings, to our knowledge, are the first to document a metabolic response of peripheral tissues to a transcriptional program emanating from the heart. While lipid metabolism is enhanced in adipose tissue of MED13cTg mice, which are lean, these mice are insulin sensitive (Grueter *et al*, 2012). Thus, their enhanced adipose lipid metabolism is not indicative of an insulin-resistant or pathological state.

Cardiac MED13 overexpression also regulates substrate oxidation in the heart. Hearts from MED13cTg mice oxidize substrate that is more readily available. In the fed state, when glucose levels are high, more glucose is oxidized, but MED13cTg hearts can still oxidize LCFA in the fasted state. Although pyruvate dehydrogenase (PDH) gene expression is decreased in MED13cTg hearts, glucose oxidation is not decreased, suggesting that other compensatory modes of PDH regulation (such as allosteric regulation by acetyl CoA, NADH, and ATP, or post-translational regulation by phosphorylation or dephosphorylation) are invoked. These results indicate that MED13cTg hearts are metabolically flexible, rather than reverting to a pathologic, glucose-dependent metabolic state.

In-depth metabolomic profiling of tissues and serum from MED13cTg mice revealed profound decreases in acylcarnitines and acyl-CoA species in heart and liver in the fed state, without changes in serum acylcarnitine species. However, we did not observe significant differences in liver and hearts from fasting mice. This data, coupled with no changes in Krebs cycle intermediary metabolites in the fed or fasted state, suggest that MED13cTg mice are metabolically flexible and display normal substrate switching from glucose to lipid metabolism in response to fasting. The metabolomic profiling also suggests an increased reliance on amino acid metabolism in the MED13TG mice to help maintain fuel homeostasis.

Although basal metabolic rates are increased in MED13cTg mice (Grueter *et al*, 2012), specifically in adipose tissue and liver, our results demonstrate that glucose and fatty acid metabolism are unchanged in skeletal muscle (Fig1). Skeletal muscle is responsible for 30–40% of resting metabolic rate (Zurlo *et al*, 1990), and consequently, lean phenotypes of many mouse models have been attributed to enhanced muscle metabolism (Reitman, 2002; Gilliam & Neufer, 2012). Furthermore, muscle can supply metabolites required for adaptation during times of nutrient stress such as fasting (Koves *et al*, 2008). Fasting serum metabolomics profiling data suggest that skeletal muscle from MED13cTg mice adapts to fasting comparably to WT muscle.

Parabiosis experiments demonstrate that circulating factors in MED13cTg mice regulate weight gain, intra-organ metabolism, and perhaps inter-organ metabolic communication (Fig6). Remarkably, the lean systemic milieu of MED13cTg mice is able to metabolically remodel WAT, liver and essentially whole-body metabolism in WT mice. Key metabolic hormones that regulate metabolism such as adiponectin, thyroxine (T4), and corticosterone are not altered in MED13cTg mice (Supplementary Fig S4). Additionally, epinephrine, norepinephrine, dopamine, and their metabolites 3,4-dihydroxyphenylacetic acid (DOPAC), 3-methoxytyramine (3-MT), and homovanillic acid (HVA) were below levels of detection in WT and MED13cTg serum, suggesting that these circulating compounds are also not responsible for the lean phenotype of the MED13cTg mouse. Furthermore, while we observed increased gene expression of brain natriuretic peptide (BNP, *Nppb*) and atrial natriuretic peptide (ANP, *Nppa*) in the ventricles of MED13cTg hearts (Supplementary Fig S3), circulating levels of ANP and BNP are most likely not critical in metabolic regulation of MED13cTg mice (Supplementary Fig S4) due to the absence of a corresponding increase in NEFA (Fig5A) (Bordicchia *et al*, 2012).

The results presented here indicate that circulating factors regulate metabolism in MED13cTg mice; however, we are still investigating specific mechanisms and molecules that are responsible for this regulation. Our data are compatible with a model in which a primary action of MED13 expression is to regulate fatty acid oxidation in the heart, resulting in secondary metabolic adaptations in liver and adipose, including increased lipid and amino acid mobilization from those depots. It is also conceivable that the heart releases metabolites that signal to peripheral tissues to control metabolism. Indeed, it was recently reported that possible metabolic crosstalk exists between heart and liver in the setting of hypertrophic cardiomyopathy (Magida & Leinwand, 2014). Such mechanisms could be mediated by yet unidentified circulating factors, including those not measured with the current targeted metabolomics approach (Fig6H).

Several components of the Mediator complex have been implicated in the regulation of metabolism. For example, MED1 regulates glucose homeostasis (Chen *et al*, 2010), and MED23 regulates insulin signaling (Wang *et al*, 2009). Several Mediator subunits regulate lipid metabolism as well, including MED15, CDK8, and cyclin C (Yang *et al*, 2006; Zhao *et al*, 2012; Zhang *et al*, 2013). MED13 is the only Mediator subunit examined thus far with respect to a potential metabolic role specifically in the heart. Thus, it will be interesting to determine whether other subunits exert similar cardiac functions and whether MED13 exerts similar metabolic functions in noncardiac tissues.

## Materials and Methods

### Animals

All animal procedures were approved by the Institutional Animal Care and Use Committee at UT Southwestern Medical Center. Animals were housed in a pathogen-free barrier facility with a 12-h light/dark cycle and maintained on standard chow (2916 Teklad Global). MED13cTg and WT littermates were generated as previously described, and backcrossed into C57Bl6 for at least 10 generations (Grueter *et al*, 2012). Male mice aged 6–8 weeks were used for all experiments except for the parabiosis experiments, and the number of animals used is specified in the figure legends. Tissue was taken in the fed state except when mentioned otherwise.

### *In vivo* [^3^H]-triolein uptake and β-oxidation

Experiments to determine tissue-specific uptake and oxidation of [^3^H]-triolein were performed as previously described (Kusminski *et al*, 2012). Briefly, [^3^H]-triolein (2 μCi per mouse in 100 μl of 5% intralipid) was injected into the mouse tail vein after a 3-h fast. Blood samples were collected at 1, 2, 5, 10, and 15 min after injection. Mice were euthanized 20 min after injection; blood samples and tissues were excised, weighed, and frozen at −80°C until processing. Lipids were extracted, and radioactivity content of blood and tissues was quantified.

### RNA deep sequencing

RNA was isolated from adipose tissue and both ventricles of WT and MED13cTg mice using TRIzol™ according to the manufacturer's instructions. Total RNA (10 ng) was submitted for transcriptome sequencing (RNA-Seq). Data analysis was performed using the software suite TopHat and Cufflink with default settings (Trapnell *et al*, 2012). Data have been deposited in NCBI's Gene Expression Omnibus and are accessible through GEO Series accession number GSE62450.

### Transmission electron microscopy

Tissues were fixed by perfusion with 4% paraformaldehyde and 1% glutaraldehyde in 0.1 M sodium cacodylate buffer. Fixed tissues were post-fixed, stained, dehydrated, and embedded in EDbed-812 resin. Tissue sections were cut and post-stained, and images were acquired on a FEI Tecnai G^2^ Spirit TEM.

### Immunoblotting

Protein was isolated from homogenized adipose tissue in modified RIPA buffer. 10 μg of protein was loaded per sample, and proteins were detected using specific antibodies.

### Histology

Liver was fixed in 4% paraformaldehyde, sectioned, and stained with oil red O.

### Mitochondrial experiments

Oxygen consumption rates (OCR) were determined using the XF24 Extracellular Flux Analyzer (Seahorse Bioscience) according to the manufacturer's instructions and as previously described (Kusminski *et al*, 2012). Briefly, mitochondria were isolated from WT and MED13cTg WAT and liver and seeded (5 μg per well) in 1× MAS buffer (70 mM sucrose, 220 mM mannitol, 10 mM KH_2_PO_4_, 5 mM HEPES, and 1 mM EDTA in 0.2% fatty-acid-free BSA). Basal OCR was measured after 10 min equilibration at 37°C. Subsequent OCR were determined after the addition of rotenone (2 μM final concentration), succinate (10 mM), antimycin A (4 μM), and ascorbate (10 mM containing 1 mM TMPD).

### Metabolomics

Serum, liver, and both heart ventricles were collected from WT and MED13cTg mice in the *ad libitum* fed state and after an overnight fast (˜18 h) and snap-frozen in liquid nitrogen until processing. Tissue was pulverized under liquid nitrogen and processed for metabolomics analysis as previously described (An *et al*, 2004; Koves *et al*, 2008).

### Langendorff heart perfusions

Hearts from WT and MED13cTg mice were quickly excised and arrested in ice-cold saline. Aortas were cannulated for retrograde perfusion with Krebs buffer supplemented with uniformly labeled long-chain fatty acids ([U-^13^C] FA, 0.1 mM, or 0.4 mM) bound to BSA and [1,6-^13^C] 10 mM or 8 mM glucose with or without insulin (10 mU/ml). A pressure transducer was placed into the left ventricle to monitor cardiac performance throughout the perfusion protocol. Hearts were perfused for 60 min and coronary flow samples taken every 15 min for oxygen consumption measurements. After 60 min of perfusion, hearts were snap-frozen in liquid nitrogen. Frozen hearts were freeze-dried, hydrated, and pulverized in 4% perchloric acid. The organic phase was collected and used for substrate utilization determination by ^13^C-NMR as previous described (Stowe *et al*, 2006).

### Hormones and other factors

Trunk blood was collected from male mice in the *ad libitum* fed state and after an overnight fast (˜18 h), and serum was used for the following measurements. Non-esterified free fatty acids (NEFA) and glucose levels were quantified using colorimetric assays (Wako Diagnostics). Thyroxine (T4) and corticosterone were measured with radioimmunoassays (RIA, MP Biomedicals), and BNP and ANP were measured with enzyme immunoassays (EIA, Sigma-Aldrich®).

### Parabiosis experiments

Male mouse littermates were surgically conjoined at 4 weeks of age. The method used was a modified protocol from Bunster and Meyer (1933) and Wright *et al* (2001). Briefly, a longitudinal incision was made on anesthetized mice from the base of the tail to just posterior to the ear, and the dorsal skin from each mouse and the ventral skin from each mouse were sutured to conjoin two mice. Isotypic and heterotypic parabiots were weighed weekly, and 7 weeks post-surgery tissues were harvested for mitochondrial isolation or snap-frozen in liquid nitrogen and processed for further analysis.

### Statistical analysis

All data are expressed as the mean ± standard error of the mean (SEM). Unpaired Student's *t*-test or two-way ANOVA with the appropriate *post hoc* test was performed to determine statistical significance, and the analysis is specified in the figure legends. A *P* < 0.05 was considered statistically significant.

## Supporting Information

for this article is available online: http://embomolmed.embopress.org

Supplementary Information

Review Process File

## References

[b1] An J, Muoio DM, Shiota M, Fujimoto Y, Cline GW, Shulman GI, Koves TR, Stevens R, Millington D, Newgard CB (2004). Hepatic expression of malonyl-CoA decarboxylase reverses muscle, liver and
whole-animal insulin resistance. Nat Med.

[b3] Bordicchia M, Liu D, Amri EZ, Ailhaud G, Dessi-Fulgheri P, Zhang C, Takahashi N, Sarzani R, Collins S (2012). Cardiac natriuretic peptides act via p38 MAPK to induce the brown fat thermogenic
program in mouse and human adipocytes. J Clin Invest.

[b4] Bunster E, Meyer RD (1933). An improved method of parabiosis. Anat Rec.

[b50] Cahill GF (1976). Starvation in man. Clin Endocrinol Metab.

[b5] Chen W, Zhang X, Birsoy K, Roeder RG (2010). A muscle-specific knockout implicates nuclear receptor coactivator MED1 in the
regulation of glucose and energy metabolism. Proc Natl Acad Sci USA.

[b6] Conaway RC, Conaway JW (2011). Function and regulation of the mediator complex. Curr Opin Genet Dev.

[b7] Gilliam LA, Neufer PD (2012). Transgenic mouse models resistant to diet-induced metabolic disease: is energy
balance the key?. J Pharmacol Exp Ther.

[b8] Grueter CE, van Rooij E, Johnson BA, DeLeon SM, Sutherland LB, Qi X, Gautron L, Elmquist JK, Bassel-Duby R, Olson EN (2012). A cardiac microRNA governs systemic energy homeostasis by regulation of
MED13. Cell.

[b9] Grueter CE (2013). Mediator complex dependent regulation of cardiac development and
disease. Genomics Proteomics Bioinformatics.

[b10] Harmancey R, Wilson CR, Taegtmeyer H (2008). Adaptation and maladaptation of the heart in obesity. Hypertension.

[b11] Karmazyn M, Purdham DM, Rajapurohitam V, Zeidan A (2008). Signalling mechanisms underlying the metabolic and other effects of adipokines on the
heart. Cardiovasc Res.

[b12] Knuesel MT, Meyer KD, Bernecky C, Taatjes DJ (2009). The human CDK8 subcomplex is a molecular switch that controls mediator coactivator
function. Genes Dev.

[b13] Koves TR, Ussher JR, Noland RC, Slentz D, Mosedale M, Ilkayeva O, Bain J, Stevens R, Dyck JR, Newgard CB (2008). Mitochondrial overload and incomplete fatty acid oxidation contribute to skeletal
muscle insulin resistance. Cell Metab.

[b14] Kusminski CM, Holland WL, Sun K, Park J, Spurgin SB, Lin Y, Askew GR, Simcox JA, McClain DA, Li C (2012). MitoNEET-driven alterations in adipocyte mitochondrial activity reveal a crucial
adaptive process that preserves insulin sensitivity in obesity. Nat Med.

[b15] Lee JH, Bassel-Duby R, Olson EN (2014). Heart- and muscle-dervied signaling system dependent on MED13 and WInless controls
obesity in *Drosophila*. Proc Natl Acad Sci USA.

[b16] Madrazo JA, Kelly DP (2008). The PPAR trio: regulators of myocardial energy metabolism in health and
disease. J Mol Cell Cardiol.

[b17] Magida JA, Leinwand LA (2014). Metabolic crosstalk between the heart and liver impacts familial hypertrophic
cardiomyopathy. EMBO Mol Med.

[b18] Pospisilik JA, Schramek D, Schnidar H, Cronin SJ, Nehme NT, Zhang X, Knauf C, Cani PD, Aumayr K, Todoric J (2010). Drosophila genome-wide obesity screen reveals hedgehog as a determinant of brown
versus white adipose cell fate. Cell.

[b19] Reitman ML (2002). Metabolic lessons from genetically lean mice. Annu Rev Nutr.

[b20] Rosen ED, Spiegelman BM (2014). What we talk about when we talk about fat. Cell.

[b22] Seiler SE, Martin OJ, Noland RC, Slentz DH, Debalsi KL, Ilkayeva OR, An J, Newgard CB, Koves TR, Muoio DM (2014). Obesity and lipid stress inhibit carnitine acetyltransferase activity. J Lipid Res.

[b23] Storlien L, Oakes ND, Kelley DE (2004). Metabolic flexibility. Proc Nutr Soc.

[b24] Stowe KA, Burgess SC, Merritt M, Sherry AD, Malloy CR (2006). Storage and oxidation of long-chain fatty acids in the C57/BL6 mouse heart as
measured by NMR spectroscopy. FEBS Lett.

[b25] Sun K, Kusminski CM, Scherer PE (2011). Adipose tissue remodeling and obesity. J Clin Invest.

[b26] Trapnell C, Roberts A, Goff L, Pertea G, Kim D, Kelley DR, Pimentel H, Salzberg SL, Rinn JL, Pachter L (2012). Differential gene and transcript expression analysis of RNA-seq experiments with
TopHat and Cufflinks. Nat Protoc.

[b27] Van Gaal LF, Mertens IL, De Block CE (2006). Mechanisms linking obesity with cardiovascular disease. Nature.

[b28] Wang W, Huang L, Huang Y, Yin JW, Berk AJ, Friedman JM, Wang G (2009). Mediator MED23 links insulin signaling to the adipogenesis transcription
cascade. Dev Cell.

[b29] Wright DE, Wagers AJ, Gulati AP, Johnson FL, Weissman IL (2001). Physiological migration of hematopoietic stem and progenitor cells. Science.

[b30] Yang F, Vought BW, Satterlee JS, Walker AK, Jim Sun ZY, Watts JL, DeBeaumont R, Saito RM, Hyberts SG, Yang S (2006). An ARC/mediator subunit required for SREBP control of cholesterol and lipid
homeostasis. Nature.

[b31] Zhang Y, Xiaoli, Zhao X, Yang F (2013). The mediator complex and lipid metabolism. J Biochem Pharmacol Res.

[b32] Zhao X, Feng D, Wang Q, Abdulla A, Xie XJ, Zhou J, Sun Y, Yang ES, Liu LP, Vaitheesvaran B (2012). Regulation of lipogenesis by cyclin-dependent kinase 8-mediated control of
SREBP-1. J Clin Invest.

[b501] Zurlo F, Larson K, Bogardus C, Ravussin E (1990). Skeletal muscle metabolism is a major determinant of resting energy
expenditure. J Clin Invest.

