# Supplementary material for: MED13-dependent signaling from the heart confers leanness by enhancing metabolism in adipose tissue and liver
Source: EMBO Mol Med. 2014 Nov 24;6(12):1610–21. doi: 10.15252/emmm.201404218 (PMC4287978; doi:10.15252/emmm.201404218)
Supplement: Supplementary file 1 — Supplementary Information [file emmm0006-1610-sd1.pdf]

EMBO Molecular Medicine

# MED13 dependent signaling from the heart confers leanness by enhancing metabolism in adipose tissue and liver

Kedryn K. Baskin, Chad E. Grueter, Christine M. Kusminski, William L. Holland, Angie L. Bookout, Santhosh Satapati, Megan Kong, Shawn C. Burgess, Craig R. Malloy, Philipp E. Scherer, Christopher B. Newgard, Rhonda Bassel-Duby and Eric N. Olson

*Corresponding author: Eric N. Olson, UT Southwestern Medical Center*

**Review timeline:**

| | |
|---|---|
| Submission date: | 01 May 2014 |
| Editorial Decision: | 21 May 2014 |
| Revision received: | 22 September 2014 |
| Editorial Decision: | 13 October 2014 |
| Revision received: | 22 October 2014 |
| Accepted: | 23 October 2014 |

## Transaction Report:

(Note: With the exception of the correction of typographical or spelling errors that could be a source of ambiguity, letters and reports are not edited. The original formatting of letters and referee reports may not be reflected in this compilation.)

*Editor: Roberto Buccione*

1st Editorial Decision 21 May 2014

Thank you for the submission of your manuscript to EMBO Molecular Medicine. We have now received comments from the three Reviewers whom we asked to evaluate your manuscript

You will see that while two Reviewers are quite appreciative of your work, one is rather negative. All considered, the (mostly overlapping) issues raised prevent us from considering publication at this time. I will not dwell into much detail as the evaluations are self-explanatory. I would like, however, to mention the main points.

Reviewer 1 is rather adamant in his/her opinion that the manuscript does not improve our understanding of the cardiac function of MED13 due to the lack of mechanistic development and therefore does not have clear elements of novelty. S/he also laments insufficient description of the fasting experiments.

Reviewer 2 is globally supportive but, again, would like to see more mechanistic insight into how MED13 stimulates white adipose tissue and liver metabolism and suggests some experimental approaches to this effect. Reviewer 2 also feels that the tenet that enhanced white adipose tissue and liver metabolism are responsible for mouse leanness is not fully supported by the observations.

Reviewer 3 is also supportive but makes a point of mentioning, similarly to the other Reviewers, the lack of any mechanistic insight into the nature of the cardiac signal that confers leanness. Reviewer 3 also lists a number of important experimental shortcomings and requests for clarification that require your action.

Clearly, all three Reviewers are of the opinion that some degree of mechanistic insight must be provided to add value and impact to your manuscript and we agree. I therefore suggest that, in addition to responding to Reviewer 3's specific points, you develop your study as far as realistically possible in a mechanistic sense for your next, revised version.

In conclusion, while publication of the paper cannot be considered at this stage, we would be happy to consider a suitably revised submission, provided that the Reviewers' concerns are addressed as outlined above with further experimentation where required.

I look forward to receiving your revised manuscript.

***** Reviewer's comments *****

Referee #1 (Comments on Novelty/Model System):

This manuscript follows-up on previously published findings that cardiac-specific MED13 overexpression confers a lean phenotype in mice. This manuscript describes a thorough metabolic characterization of the MED13 transgenic mouse and confirms the previous finding of systemically altered metabolic homeostasis. However, these data offer modest mechanistic insight beyond what was described in the 2012 Cell paper. While there is a wealth of data, and the characterization of the mouse should be published somewhere, it does not seem to me to have substantial new insight into the cardiac MED13 effect.

Referee #1 (Remarks):

Reported here is a thorough evaluation of the transgenic overexpressor mouse. The studies are certainly extensive and well done, by expert investigators, and the integrated nature of metabolism can make metabolic effects difficult to pinpoint. However, mechanistic insight in this extensive characterization beyond the previous 2012 Cell paper is relatively modest, and the direct potential connection to the heart remains unclear. The authors describe major possibilities in their discussion; it seems that they are aware that their study has not really unraveled the effect to any significant degree.

The data demonstrate that WAT of the transgenic mouse has increased lipid uptake, mitochondrial activity and energy expenditure. However, no mechanism for this effect by cardiac MED13 is described.

It is not clear how the deep sequencing in the heart added beyond the previous microarray study.

There is no description of how long the animals were fasted for. A longer fast and/or a re-feeding experiment could have allowed a stronger conclusion on metabolic flexibility of the transgenic heart.

Referee #2 (Comments on Novelty/Model System):

This is a follow up study of the author's work published in Cell. In this study, they investigated the mechanism by which the heart specific expression of MED13 prominently mediate the lean phenotype. Although the study is descriptive in nature, it clearly demonstrates that the heart specific expression of MED13 affects the metabolic phenotype in the extra-cardiac energy depots. The demonstration of such metabolic communication from the heart to the peripheral organs has been rarely done in the past. In addition, the authors show that the heart in MED13cTg is metabolically flexible, which would add some therapeutic implications to the study. I believe that the authors' findings are novel and important.

Referee #2 (Remarks):

The authors investigated the mechanism by which transgenic mice with heart-specific expression of MED13 induces changes in systemic energy homeostasis, using the state-of-the-art metabolic analyses including metabolomics. Interestingly, although MED13 in the heart decreases metabolic gene expression and metabolite levels in heart and liver, it enhances them in WAT. It appears that MED13 in the heart has an ability to promote systemic energy expenditure in extra-cardiac energy depots.

This is a follow up study of the author's work published in Cell. In this study, they investigated the mechanism by which the heart specific expression of MED13 prominently mediates the lean phenotype. Although the study is descriptive in nature, it clearly demonstrates that the heart specific expression of MED13 affects the metabolic phenotype in the extra-cardiac energy depots. In addition, the authors show that the heart in MED13cTg is metabolically flexible, which would add some therapeutic implications to the study.

The authors discussed potential mechanisms by which cardiac MED13 stimulates metabolic rate of WAT and liver. This is a central question in this work but the authors do not address this question experimentally in this work. Ideally, the authors should include at least some experiments to address this issue. For example, the authors could have shown whether or not the serum in the MED13 mice contains factors inducing metabolic changes in WAT and liver.

Specific
Does MED13 show cell autonomous effect in cultured cardiomyocytes and reproduce the effect in Tg?

The authors do not prove yet that the enhanced metabolism in WAT and liver is really responsible for the leanness of the MED13cTg. Thus, the title of the paper should be modified.

Referee #3 (Remarks):

This manuscript's sequencing and metabolomics of the affected extra-cardiac tissues in the cardiac-specific MED13 transgenic mouse model provides a compelling extension of the group's previous characterization of this mouse [Grueter et al., Cell, 2012]. The manuscript shows that the expression of a heart-specific transgene results in elevated lipid oxidation in white adipose and liver tissues. These findings may account for the protection from high-fat diet feeding and metabolic syndrome conferred by cardiac MED13 expression previously observed [Grueter et al., Cell, 2012]. Baskin and colleagues offer valuable insight into an evolving role for the heart in regulating systemic metabolism, but falls short in describing inter-organ signaling moieties.

The manuscript is strong but in need of some clarification and experiments.

1. The most notable issue is the absence of data or a hypothesis regarding the means of cardiac regulation or the nature of the "signal from the heart [that] confers leanness." The authors offer 3 possible explanations for the "signal", which seem plausible, and even interrelated, however there is little data to support these hypotheses.
(A) A factor secreted [from the heart], such as natriuretic peptides; The Olson group previously found a >50% reduction in cardiac ANF expression in MED13 transgenic mice [Fig.5G, Grueter et al., Cell, 2012]. Therefore, we recommend qPCR analysis of cardiac ANF/BNP transcript levels, as well circulating levels of ANF/BNP in both the fasted and fed states to provide support for or against this claim.
(B) A "circulating hormonal factor." It appears as though 2 major adipose-derived hormone peptides, adiponectin and leptin, can be ruled out since Grueter el al. reported unaltered food consumption and circulating leptin levels, and Baskin et al. reported unaltered adiponectin levels in the supplement (SF4E). Therefore, it would be good for the authors to measure circulating catecholamines or other hormone candidates before making this suggestion. However, adrenergic agonism of cardiac myocytes results in the repression of nuclear receptor activity, particularly the downregulation of Cpt1b [Barger, JCI, 2000], which is upregulated more than 2-fold in the MED13 transgenic heart.
(C) Alternatively, the authors stated that the cardiac impact of MED13 "necessitates mobilization of peripheral energy reserves" by repressing nuclear receptor activity and describe this as increased "metabolic flexibility." Please identify the affected nuclear receptors.
While there is limited data to infer increased flexibility (Fig 4E/F, H/I), the heart of the MED13 transgenic mouse appears to have made a pathological glycolytic shift away from fatty acid metabolism, as suggested by decreases in

absolute or relative fatty acid oxidation in the fed and fasted states, respectively, and an increase in both absolute b-myosin expression and the apparent β:α myosin ratio [Fig.5E-F, Grueter, et al., Cell, 2012]. Do the authors believe that increased reliance upon glucose utilization would "necessitate" increased glucose availability? This point should be discussed.

2. How do the author's reconcile an approximately 2-fold increase in Cpt1β expression and decreased Cpt2 expression (Fig.4A) with an almost universal decrease in acyl carnitines (Supp. Table 3) in the fed MED13 heart? Is this due to the downregulation of Acsl1 and a large-scale decrease in the upstream myocardial acyl-CoA pool (Fig.4A; Supp Table 4)? If acyl-CoA synthase expression is disrupted and radiolabeled triolein uptake is normal, if not improved in the MED13 transgenic heart (Fig.1B), wouldn't it be good to assess the fatty acid compositions of the triglyceride and non-esterified fatty acid (non-acyl CoA ester) pools? Also, how do the authors reconcile normal radiolabeled triolein oxidation in vivo (Fig. 1C) with reduced oxidation of some other unspecified (different?) long chain fatty acid in the MED13 transgenic heart in a subsequent figure (Fig. 4F)?

3. Have the authors measured fasted serum glucose, non-esterified fatty and triglyceride levels in the fasted MED13 mouse? If so, do these mice display any change in circulating non-esterified fatty acids that would conform to normalized myocardial long-chain fatty acid oxidation rates or increased natriuretic peptide secretion in the fasted state?
What is the nature of the shift towards a glucose preference in the MED13 heart? (A) Are glycolytic genes upregulated in the fasted or fed MED13 heart? (B) How do the authors explain increased glucose oxidation, despite decreased pyruvate dehydrogenase expression (Fig.4A,J)? (C) Are the substrates for increased endogenous myocardial glucose oxidation in the fasted state derived from glycogenolysis?

4. Wouldn't decreased lipid storage or increased oxidation in adipose and liver result in reduced fasting plasma non-esterified fatty levels and triglyceride-rich lipoprotein levels, respectively? Neither the heart nor liver from MED13 transgenics show any indication of increased long-chain fatty acyl CoA content in the fasted state.
Are the differences in cardiac acyl carnitines (e.g. C16:0 and C18:0) between wildtype and transgenic mice in the fasted state significantly different?

5. Please briefly discuss the increases in both (fed) cardiac and hepatic propionyl CoA content, and the presence of increased substrate, such as ketogenic amino acids (methionine, valine, isoleucine) and product (succinyl CoA) in the heart, but not elevated or depleted in liver.

6. The measurement of hepatic and cardiac mitochondrial oxygen consumption (Fig.3B, 4E,H) should be repeated with a long-chain fatty acid or acyl CoA, rather than just electron transport chain substrates. This assay should also be performed on mitochondria isolated from adipose tissue.

7. Why does increased lipid oxidation (Fig. 1C, 3B) not result in any change in Krebs cycle intermediates in the livers of transgenic mice (Supp Fig. 1A)? Is it as simple as increased flux? Please provide an analysis of fatty acid oxidation and Krebs cycle gene mRNA levels in fed and fasted livers.

8. Please address organ-specific propionyl and acetyl CoA profiles. Are proportional increases in both propionyl and acetyl CoA suggestive of increased odd-chain fatty acid oxidation in the MED13 heart? Is there no subsequent change in succinate or citrate levels due to decreased methylmalonyl CoA mutase or citrate synthase, respectively, or a general decrease in Krebs cycle gene expression? Are these genes elevated in the livers of MED13 transgenics?

Minor points/questions
1. Validate pertinent differences in transcript levels determined by sequencing, including Cpt1/2, fatty acid oxidation genes, and Pdh with qPCR.
2. Also measure myocardial Cpt1α transcript levels.
3. Why is there increased lipid uptake or oxidation in some highly metabolically active extra-cardiac tissues, such as adipose and liver, but not others (e.g. skeletal muscle)? Does this imply something about the identity or activity of the "circulation hormonal factor" associated with the extra-cardiac MED13 Tg phenotype?
4. Were experiments on cardiac tissue from both ventricles or the left ventricle?

5. What was the utility of a ceramide analysis?
6. Why were wildtype and transgenic serum glucose levels so high (nearly 200mg/dl)?

1st Revision - authors' response 22 September 2014

Referee #1 (Comments on Novelty/Model System):

*This manuscript follows-up on previously published findings that cardiac-specific MED13 overexpression confers a lean phenotype in mice. This manuscript describes a thorough metabolic characterization of the MED13 transgenic mouse and confirms the previous finding of systemically altered metabolic homeostasis. However, these data offer modest mechanistic insight beyond what was described in the 2012 Cell paper. While there is a wealth of data, and the characterization of the mouse should be published somewhere, it does not seem to me to have substantial new insight into the cardiac MED13 effect.*

Thank you for your suggestions to improve our manuscript. We agree that the original submission of our paper was primarily descriptive. While identification of the exact mechanism responsible for the lean phenotype of the MED13cTg mice is beyond the scope of the current manuscript, we have performed additional experiments which address a general mechanism. These data are included in the revised manuscript and the Discussion has been rewritten.

Referee #1 (Remarks):

*Reported here is a thorough evaluation of the transgenic overexpressor mouse. The studies are certainly extensive and well done, by expert investigators, and the integrated nature of metabolism can make metabolic effects difficult to pinpoint. However, mechanistic insight in this extensive characterization beyond the previous 2012 Cell paper is relatively modest, and the direct potential connection to the heart remains unclear. The authors describe major possibilities in their discussion; it seems that they are aware that their study has not really unraveled the effect to any significant degree.*

We have performed additional experiments which have revealed the nature of systemic metabolic regulation in the MED13cTg mouse (see Figure 6 and section entitled "Circulating factor(s) regulate enhanced WAT and liver metabolism and contribute to the lean phenotype of MED13cTg mice"). As you mentioned, the Discussion of the original submission described possible mechanisms regulating enhanced metabolic rate and leanness in this model.

To further define the mechanism responsible for leanness in response to cardiac MED13 expression, we have performed mouse parabiosis experiments and subsequent gene expression analysis and metabolic measurements demonstrate that circulating factor(s) in MED13cTg mice are responsible for the lean phenotype. We consider these experiments to be a major advancement in our understanding of metabolic regulation of these mice. We measured catecholamines and several hormones that have previously been shown to regulate metabolism; however, none seem to be involved in regulating metabolism in MED13cTg mice. Although we are actively working to identify other circulating factor(s) regulating leanness, we think these experiments are beyond the scope of the current manuscript.

*The data demonstrate that WAT of the transgenic mouse has increased lipid uptake, mitochondrial activity and energy expenditure. However, no mechanism for this effect by cardiac MED13 is described.*

As mentioned above, the parabiosis experiments demonstrate that circulating factors regulate increased energy expenditure and mitochondrial activity. We are taking many different avenues to identify specific factor(s) responsible for enhanced metabolism, but we think these experiments are beyond the scope of the current manuscript.

*It is not clear how the deep sequencing in the heart added beyond the previous microarray study.*

In this study we specifically looked at the ventricles of mature (8wk old) male MED13cTg mice. Although not all genes are discussed within this manuscript, RNAseq analysis, compared to microarray analysis, gives a much more detailed picture of the genes regulated by MED13 in the heart.

*There is no description of how long the animals were fasted for. A longer fast and/or a re-feeding experiment could have allowed a stronger conclusion on metabolic flexibility of the transgenic heart.*

The animals were fasted overnight 18 hours) and the tissues were collected in the late afternoon the following day. This has now been clarified in the materials and methods sections under "Metabolomics".

Referee #2 (Comments on Novelty/Model System):

*This is a follow up study of the author's work published in Cell. In this study, they investigated the mechanism by which the heart specific expression of MED13 prominently mediate the lean phenotype. Although the study is descriptive in nature, it clearly demonstrates that the heart specific expression of MED13 affects the metabolic phenotype in the extra-cardiac energy depots. The demonstration of such metabolic communication from the heart to the peripheral organs has been rarely done in the past. In addition, the authors show that the heart in MED13cTg is metabolically flexible, which would add some therapeutic implications to the study. I believe that the authors' findings are novel and important.*

Thank you for your enthusiasm regarding our study. We appreciate your understanding of the difficult nature of investigating metabolic communication between organs. Because our new data included in the revision strongly support our initial hypothesis of inter-organ communication within the MED13cTg mouse model, we hope you find our manuscript to be more mechanistic in nature.

Referee #2 (Remarks):

*The authors investigated the mechanism by which transgenic mice with heart-specific expression of MED13 induces changes in systemic energy homeostasis, using the state-of-the-art metabolic analyses including metabolomics. Interestingly, although MED13 in the heart decreases metabolic gene expression and metabolite levels in heart and liver, it enhances them in WAT. It appears that MED13 in the heart has an ability to promote systemic energy expenditure in extra-cardiac energy depots.*

*This is a follow up study of the author's work published in Cell. In this study, they investigated the mechanism by which the heart specific expression of MED13 prominently mediates the lean phenotype. Although the study is descriptive in nature, it clearly demonstrates that the heart specific expression of MED13 affects the metabolic phenotype in the extra-cardiac energy depots. In addition, the authors show that the heart in MED13cTg is metabolically flexible, which would add some therapeutic implications to the study.*

*The authors discussed potential mechanisms by which cardiac MED13 stimulates metabolic rate of WAT and liver. This is a central question in this work but the authors do not address this question experimentally in this work. Ideally, the authors should include at least some experiments to address this issue. For example, the authors could have shown whether or not the serum in the MED13 mice contains factors inducing metabolic changes in WAT and liver.*

Thank you for your suggestions. We have now included in vivo experiments demonstrating that circulating factors regulate enhanced metabolic rates in WAT and liver, which ultimately are responsible for decreased weight gain (discussed in detail below). We are excited about the results of these experiments and consider these data to be a major advancement in our understanding of the metabolic regulation of the MED13cTg mouse.

*Specific*

*Does MED13 show cell autonomous effect in cultured cardiomyocytes and reproduce the effect in Tg?*

Given the intricacies of inter-organ communication in vivo, we addressed this issue in a more physiological setting. We asked the question, "Does a circulating factor in the MED13cTg mouse regulate metabolism in extra-cardiac energy depots?" We performed parabiosis experiments in WT-WT, Tg-Tg, and WT-Tg mice

and found that circulating factor(s) in MED13cTg mice are responsible for the lean phenotype. Specifically, circulating factors in MED13cTg mice increase metabolic rates in WAT and liver in conjoined WT mice leading to decreased weight gain in WT mice (see Figure 6 and section "Circulating factor(s) regulate enhanced WAT and liver metabolism and contribute to the lean phenotype of MED13cTg mice").

*The authors do not prove yet that the enhanced metabolism in WAT and liver is really responsible for the leanness of the MED13cTg. Thus, the title of the paper should be modified.*

Thanks for the suggestion. We think the parabiosis experiments support our conclusion that the lean phenotype of the MED13cTg mouse occurs through regulation of WAT and liver metabolism. However, if you disagree, we will make the appropriate change.

Referee #3 (Remarks):

*This manuscript's sequencing and metabolomics of the affected extra-cardiac tissues in the cardiac-specific MED13 transgenic mouse model provides a compelling extension of the group's previous characterization of this mouse [Grueter et al., Cell, 2012]. The manuscript shows that the expression of a heart-specific transgene results in elevated lipid oxidation in white adipose and liver tissues. These findings may account for the protection from high-fat diet feeding and metabolic syndrome conferred by cardiac MED13 expression previously observed [Grueter et al., Cell, 2012]. Baskin and colleagues offer valuable insight into an evolving role for the heart in regulating systemic metabolism, but falls short in describing inter-organ signaling moieties.*

Thanks for your careful reading and thorough assessment of our manuscript. We agree that the original version of our manuscript required additional experiments to more clearly understand inter-organ metabolic crosstalk in MED13cTg mice. We have performed additional experiments that demonstrate metabolic regulation of WAT and liver by circulating factors. We consider these experiments to be a major advancement in our understanding of metabolic regulation of MED13cTg mice, and hope you find these experiments compelling as well.

*The manuscript is strong but in need of some clarification and experiments.*

Thank you for pointing out these issues. We have addressed these matters in detail below.

*1. The most notable issue is the absence of data or a hypothesis regarding the means of cardiac regulation or the nature of the "signal from the heart [that] confers leanness." The authors offer 3 possible explanations for the "signal", which seem plausible, and even interrelated, however there is little data to support these hypotheses.*

We have addressed these issues below and have modified the Discussion of the manuscript accordingly.

*(A) A factor secreted [from the heart], such as natriuretic peptides; The Olson group previously found a >50% reduction in cardiac ANF expression in MED13 transgenic mice [Fig.5G, Grueter et al., Cell, 2012]. Therefore, we recommend qPCR analysis of cardiac ANF/BNP transcript levels, as well circulating levels of ANF/BNP in both the fasted and fed states to provide support for or against this claim.*

Thank you for this suggestion. We have now measured ANF/BNP transcript levels in ventricles from fed and fasted mice and circulating levels in fed and fasted mice. We previously found decreased (although not significant) levels of ANF gene expression in whole hearts of MED13cTg mice. We have measured ANF and BNP in MED13cTg ventricles by RNAseq and qPCR and we observed an increase in the expression of both genes in both the ventricles of MED13cTg mice. Expression of ANF and BNP in the ventricles is not affected by fasting. These data are now included in Figure E3C. We also analyzed circulating levels of ANP and BNP in fed and fasted MED13cTg mice. BNP circulating levels were not significantly different between WT and MED13cTg mice in the fed state. Circulating BNP levels were higher in MED13cTg fasted mice compared to WT fasted mice, albeit only slightly (Figure E4G). Although circulating ANP levels trended to be higher in MED13cTg mice, this was not statistically different (Figure E4H). These data have now been added to the discussion.

*(B) A "circulating hormonal factor." It appears as though 2 major adipose-derived hormone peptides, adiponectin and leptin, can be ruled out since Grueter el al. reported unaltered food consumption and circulating leptin levels, and Baskin et al. reported unaltered adiponectin levels in the supplement (SF4E). Therefore, it would be good for the authors to measure circulating catecholamines or other hormone candidates before making this suggestion. However, adrenergic agonism of cardiac myocytes results in the repression of nuclear receptor activity, particularly the downregulation of Cpt1β [Barger, JCI, 2000], which is upregulated more than 2-fold in the MED13 transgenic heart.*

This was an excellent suggestion. In collaboration with Vanderbilt University Neurochemistry Core we have measured a panel of circulating catecholamines. Among the catecholamines measured, many were below the detection level in fed and fasted serum collected from WT and MED13cTg mice. These include norepinephrine, epinephrine, dopamine and its metabolites 3,4-dihydroxyphenylacetic acid (DOPAC), 3-methoxytyramine ( 3-MT) and homovanillic acid (HVA). While there is no data to show due to such low circulating levels, we have referred to these results in the Discussion. We also saw no differences in corticosterone or the thyroid hormone thyroxine (T4) (Figure E4). Thus, we conclude that circulating factors regulate metabolism in the MED13cTg mouse (demonstrated through parabiosis experiments in Figure 6). While we have not yet identified these factor(s), they are likely not catecholamines.

We also apologize because we mis-calculated the fold change in Cpt1b expression in the heart. This has been corrected.

*(C) Alternatively, the authors stated that the cardiac impact of MED13 "necessitates mobilization of peripheral energy reserves" by repressing nuclear receptor activity and describe this as increased "metabolic flexibility." Please identify the affected nuclear receptors.*

We previously demonstrated that MED13 represses expression of genes regulated by the following nuclear receptors PPARg, thyroid hormone receptor, RXR, and LXR (Grueter et al., 2012). In our RNAseq analysis we found decreased expression of PPARGC1a and PPARa in MED13cTg ventricles.

*While there is limited data to infer increased flexibility (Fig 4E/F, H/I), the heart of the MED13 transgenic mouse appears to have made a pathological glycolytic shift away from fatty acid metabolism, as suggested by decreases in absolute or relative fatty acid oxidation in the fed and fasted states, respectively, and an increase in both absolute β-myosin expression and the apparent β:α myosin ratio [Fig.5E-F, Grueter, et al., Cell, 2012]. Do the authors believe that increased reliance upon glucose utilization would "necessitate" increased glucose availability? This point should be discussed.*

Our experiments demonstrate that MED13cTg hearts oxidize less LCFA in the fed state, but the same amount of LCFA in the fasted state. Furthermore, there are no significant differences in glucose oxidation in MED13cTg hearts in the fed or fasted state. These data suggest that even though MED13cTg hearts prefer glucose in the fed state (the predominant substrate in the fed state), when LCFA are the dominant available substrate, MED13cTg hearts can still function normally. The metabolomics data also support this conclusion. Acyl-CoAs and Acylcarnitines are decreased in the MED13cTg heart in the fed state compared to WT hearts, but in the fasted state there is no difference between WT and MED13cTg hearts. Because FAO is decreased and not completely shut off in MED13cTg hearts when perfused in the fed state, and FAO is unaltered in the fasted state, we do not think this is reminiscent of a pathological shift in substrate utilization in the heart.

Given evidence for increased amino acid-derived metabolites in non-cardiac tissues in the Med13 mice, we cannot conclude that an increase in glucose availability is necessarily required to sustain metabolism in the fed state, as amino acids could also contribute.  A precise answer to this question would require isotope dilution/clamp studies, which we feel are beyond the scope of the study.

*2. How do the author's reconcile an approximately 2-fold increase in Cpt1β expression and decreased Cpt2 expression (Fig.4A) with an almost universal decrease in acyl carnitines (Supp. Table 3) in the fed MED13 heart?*

As mentioned above, Cpt1b expression was incorrectly reported and has now been corrected.

*Is this due to the downregulation of Acsl1 and a large-scale decrease in the upstream myocardial acyl-CoA pool (Fig.4A; Supp Table 4)? If acyl-CoA synthase expression is disrupted and radiolabeled triolein uptake is normal, if*

*not improved in the MED13 transgenic heart (Fig.1B), wouldn't it be good to assess the fatty acid compositions of the triglyceride and non-esterified fatty acid (non-acyl CoA ester) pools?*

This is a good suggestion. Although it would be interesting to understand all details of metabolism in the MED13cTg heart, we think measuring compositions of triglyceride and non-acyl CoA ester pools is beyond the scope of the current study.

*Also, how do the authors reconcile normal radiolabeled triolein oxidation in vivo (Fig. 1C) with reduced oxidation of some other unspecified (different?) long chain fatty acid in the MED13 transgenic heart in a subsequent figure (Fig. 4F)?*

Thanks for bringing up this point. We don't think comparing figure 1 data with figure 4F is the appropriate comparison. The triolein uptake and oxidation measurements in vivo are calculated 20 minutes after tail vein delivery in the fasted-like state (Fig. 1). In isolated heart perfusions oxidation rates of long-chain fatty acids are similar in WT and MED13cTg hearts in the fasted-like state (Fig. 4I).

*3. Have the authors measured fasted serum glucose, non-esterified fatty and triglyceride levels in the fasted MED13 mouse? If so, do these mice display any change in circulating non-esterified fatty acids that would conform to normalized myocardial long-chain fatty acid oxidation rates or increased natriuretic peptide secretion in the fasted state?*

We have already measured fed/fasted serum glucose (Fig. 5C; previously Supplemental Fig. 4D) and we have added fed/fasted serum NEFA and triglycerides (Fig. 5A and 5B). There is no significant difference in glucose, triglycerides, or NEFA in MED13cTg serum in the fed or fasted state, but there is the expected decrease in glucose and triglycerides, and increase in NEFA in the fasted state in WT and MED13cTg serum. Although we found slightly elevated circulating BNP levels in fasted serum from MED13cTg mice (Figure E4I), this is not accompanied by increased NEFA when compared to fasted serum from WT mice (Figure 5A).

*What is the nature of the shift towards a glucose preference in the MED13 heart? (A) Are glycolytic genes upregulated in the fasted or fed MED13 heart?*

We have only measured glucose and glycogen metabolism genes in ventricles collected from animals in the ad libitum fed state by RNA-seq. The majority of these genes are decreased in MED13cTg ventricles. Because we don't see a difference in glucose oxidation per se, we did not investigate this further.

*(B) How do the authors explain increased glucose oxidation, despite decreased pyruvate dehydrogenase expression (Fig.4A,J)?*

Glucose oxidation per se is unchanged in MED13cTg hearts, suggesting that other compensatory modes of PDH regulation (E.G. allosteric regulation by acetyl CoA, NADH, and ATP, or post-translational regulation by phosphorylation/dephosphorylation) are invoked. This point has been added in the discussion. While it is interesting that we see many genes downregulated in the MED13cTg heart, ultimately the oxidation data is a more functional readout of what is occurring on a metabolic level.

*(C) Are the substrates for increased endogenous myocardial glucose oxidation in the fasted state derived from glycogenolysis?*

Unfortunately, we are unable to determine which specific endogenous substrates are oxidized in the heart perfusion experiments given the nature of the NMR measurements, and thus we cannot definitively say that endogenous myocardial glucose oxidation is increased in MED13cTg hearts in the fed state (Fig. 4I). While it would be interesting to investigate which endogenous substrates are utilized, we would need to repeat the heart perfusion experiments with all possible labeled substrates, which we feel is beyond the scope of the current study.

*4. Wouldn't decreased lipid storage or increased oxidation in adipose and liver result in reduced fasting plasma non-esterified fatty levels and triglyceride-rich lipoprotein levels, respectively? Neither the heart nor liver from MED13 transgenics show any indication of increased long-chain fatty acyl CoA content in the fasted state.*

This is a good point, and one which we think supports our conclusion that MED13cTg mice are more metabolically flexible than WT mice. Triglycerides are slightly decreased (although not significant) in

serum from MED13cTg mice in the fed state and we expected to see decreased fasting triglycerides in MED13cTg serum, compared to fasted WT serum, but this is not the case (see Fig. 5B). Therefore, even though lipid metabolism is greatly enhanced in MED13cTg mice, they maintain the ability to fine tune their metabolism/metabolic rates when substrate supply is decreased, such as during fasting. This is further supported by fed/fasted NEFA levels (Figure 5A).

*Are the differences in cardiac acyl carnitines (e.g. C16:0 and C18:0) between wildtype and transgenic mice in the fasted state significantly different?*

We did not see statistical differences in the fasted state when comparing WT and MED13cTg acyl carnitines in the heart. For these specific examples, C16:0 and C18:0, the respective p values are 0.39 and 0.295.

*5. Please briefly discuss the increases in both (fed) cardiac and hepatic propionyl CoA content, and the presence of increased substrate, such as ketogenic amino acids (methionine, valine, isoleucine) and product (succinyl CoA) in the heart, but not elevated or depleted in liver.*

We agree that these are interesting data. We attribute the differences in amino acid levels in liver and heart as evidence of amino acid mobilization and utilization that contribute to maintenance of metabolic homeostasis and normal levels of Krebs cycle intermediates in MED13cTg mice.

In the original submission we discussed these data in the section titled "MED13cTg mice maintain the ability to adapt metabolically to fasting", in the 3rd paragraph.

"Amino acid profiling revealed increases in the branched chain amino acids valine, leucine, and isoleucine, and neutral amino acids, proline and methionine in heart samples from fed MED13cTg mice compared to fed WT mice; however, these metabolites were not different in hearts from the two strains in the fasted state (Figure 5I). These same amino acids were elevated in serum of fed MED13cTg compared to fed WT mice (Figure 5G). Changes in these metabolites were not evident between strains in liver in the fed or fasted states, but the urea cycle intermediate ornithine was elevated in fed MED13cTg mice compared to fed WT mice (Figure 5H). Interestingly, propionyl CoA, a product of methionine, valine, and isoleucine catabolism, was elevated in heart (Figure E4A, Expanded View Table 2) and liver (Figure E4B, Expanded View Table 4) of fed MED13cTg mice compared to fed WT mice. Additionally, C5 acylcarnitine, also a product of BCAA catabolism, was increased in plasma of MED13cTg mice (Figure 5C). These findings may suggest that amino acid mobilization and utilization contribute to maintenance of metabolic homeostasis and normal levels of Krebs cycle intermediates in MED13cTg mice."

*6. The measurement of hepatic and cardiac mitochondrial oxygen consumption (Fig.3B, 4E,H) should be repeated with a long-chain fatty acid or acyl CoA, rather than just electron transport chain substrates. This assay should also be performed on mitochondria isolated from adipose tissue.*

Because the in vivo experiments show drastic changes in triolein oxidation, we wanted to determine whether enhanced oxidation was due to enhanced mitochondrial oxidative capacity. The mitochondrial oxygen consumption rates in Fig. 3B are taken using the Seahorse Bioanalyzer with an assay designed to probe electron flow through the electron transport chain, and thus uses appropriate substrates (see Rogers GW et a., PLos One, 2011). We have performed these experiments on mitochondria isolated from adipose tissue and liver of WT and MED13cTG mice in the parabiosis experiments. Indeed, mitochondria in WAT from isochronic TG parabiots have significantly higher oxygen consumption rates than isochronic WT parabiots. These new experiments are included in the revised version of the manuscript (Figure 6). While these experiments could be repeated again with additional substrates (e.g. long-chain fatty acids), we do not think this additional data would change our conclusions.

*7. Why does increased lipid oxidation (Fig. 1C, 3B) not result in any change in Krebs cycle intermediates in the livers of transgenic mice (Supp Fig. 1A)? Is it as simple as increased flux? Please provide an analysis of fatty acid oxidation and Krebs cycle gene mRNA levels in fed and fasted livers.*

You raise an interesting point. We discuss these data in the section entitled "MED13cTg mice maintain the ability to adapt metabolically to fasting", in the 2nd paragraph.

Lipid oxidation is increased in MED13cTg liver, which leads to decreased accumulation of acylcarnitine and acyl-CoA (medium/long/very long- chain) species (Figure 3). "Krebs Cycle intermediates were not different in MED13cTg compared to WT mice in either the fed or fasted states, in heart or in liver (Figure

E2A and E2B). This suggests that despite the large decreases in lipid-derived acylcarnitine and acyl-CoA intermediates, substrate influx to the Krebs cycle remains adequate to maintain normal levels of all intermediates."

Although we did not directly test this hypothesis, we agree with the referee that Krebs cycle intermediates are not different in MED13cTg livers due to flux through the cycle. We have now provided the referee with FAO and Krebs cycle gene expression analysis in fed/fasted WT/TG liver (see below). There were no significant changes in gene expression in liver, neither in the fed state (first graph below), nor when comparing fold change in the fed/fasted state (second graph below).

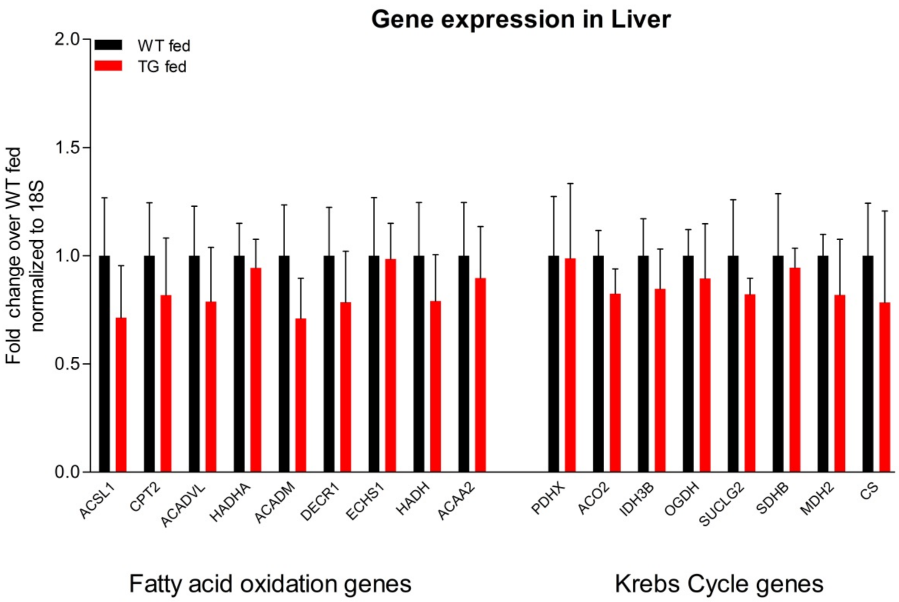


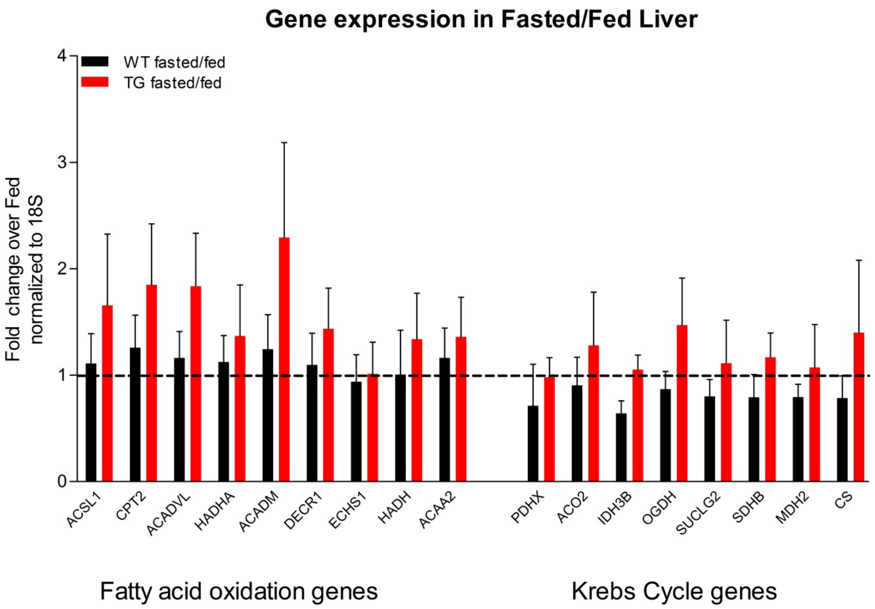

*8. Please address organ-specific propionyl and acetyl CoA profiles. Are proportional increases in both propionyl and acetyl CoA suggestive of increased odd-chain fatty acid oxidation in the MED13 heart? Is there no subsequent change in succinate or citrate levels due to decreased methylmalonyl CoA mutase or citrate synthase, respectively, or a general decrease in Krebs cycle gene expression?*

We discussed the difference in acyl CoA profiles in the original submission (see point #5 above). We think increases in CoA pools in Med13cTg heart and liver in the fed state could suggest that amino acid mobilization and utilization contribute to maintenance of metabolic homeostasis and normal levels of Krebs cycle intermediates in MED13cTg mice, and that this is independent of Krebs Cycle gene expression.

*Are these genes elevated in the livers of MED13 transgenics?*

These genes are not altered in MED13cTg liver. For gene expression data please see graphs and discussion in point 7 above.

*Minor points/questions*

*1. Validate pertinent differences in transcript levels determined by sequencing, including Cpt1/2, fatty acid oxidation genes, and Pdh with qPCR.*

We have now included this data in Supplemental Figure 1 and 3B.

*2. Also measure myocardial Cpt1α transcript levels.*

We have now included this data in Supplemental Figure 1 and 3B.

*3. Why is there increased lipid uptake or oxidation in some highly metabolically active extra-cardiac tissues, such as adipose and liver, but not others (e.g. skeletal muscle)? Does this imply something about the identity or activity of the "circulation hormonal factor" associated with the extra-cardiac MED13 Tg phenotype?*

The referee brings up an interesting point, and this is the exact line of thinking we have. We are currently investigating circulating factors in MED13cTg mice, but we think this is beyond the scope of the current manuscript.

*4. Were experiments on cardiac tissue from both ventricles or the left ventricle?*

All experiments were performed on both ventricles from the heart, and this now been clarified in the materials and methods section.

*5. What was the utility of a ceramide analysis?*

Ceramides are bioactive lipids that can regulate a number of physiological functions, and have been implicated in obesity-related complications. Also ceramides are derived from palmitate, and thus serve as a means of following a potentially important metabolic fate of a major saturated fatty acid in our model. We quantified ceramides to determine whether they might be contributing to regulation of metabolism in liver and heart of MED13cT mice (elegantly reviewed by Bikman and Summers, JCI 2011).

*6. Why were wildtype and transgenic serum glucose levels so high (nearly 200mg/dl)?*

The serum glucose levels were slightly higher than expected. We attribute this to the manner in which blood was drawn for the experiments in supplemental figure 3A. We collected blood by mandibular bleed which is a relatively slower collection technique and is not stress-free, which can raise blood glucose levels.

2nd Editorial Decision 13 October 2014

Thank you for the submission of your revised manuscript to EMBO Molecular Medicine. We have now received the enclosed reports from the referees that were asked to re-assess it. As you will see the reviewers are now supportive and I am pleased to inform you that we will be able to accept your manuscript pending the following final amendment:

1) Please consider Reviewer 1's comments on the nomenclature to be used for the parabiosis experiment and amend the text accordingly.

Please submit your revised manuscript within two weeks. I look forward to seeing a revised form of your manuscript as soon as possible.

***** Reviewer's comments *****

Referee #1 (Comments on Novelty/Model System):

My comments were suitably addressed, with one minor thing for the authors to consider.

Referee #1 (Remarks):

I am satisfied by the responses, with one minor point:

The authors may want to consider that, although the nomenclature is not definitively established by any organization, it is most common among parabiosis investigators to use "isotypic" for mice of same genotype, and "heterotypic" for mice of different genotypes.

It is most common to use "isochronic" for mice of same age joined together, regardless of genotype, and "heterochronic" for mice of different ages, regardless of genotype.

So, given your experiments of mice of the same age but different genotypes joined in parabiosis, you might want to use the terms isochronic isotypic pairs and isochronic heterotypic pairs. (though many would simply drop the isochronic and just call them isotypic and heterotypic)

Referee #2 (Comments on Novelty/Model System):

In this revision, the authors have conducted an important experiment demonstrating that circulating factors regulate enhanced metabolic rates in WAT and liver, which ultimately are responsible for decreased weight gain. Overall, the authors' findings are novel, the experiments are well done, and the manuscript is well written.

Referee #3 (Remarks):

The authors have done a great deal of additional work. While the primary question of mechanism remains, this is signficant in potential impact. The parabiosis experiment is appreciatively difficult to perform and excludes some role of the nervous system in the observed phenotype. Future work will likely elucidate the mechanism by which the transgenic heart affects extra-cardiac tissues.

2nd Revision - authors' response 22 October 2014

Referee #1 (Comments on Novelty/Model System):
My comments were suitably addressed, with one minor thing for the authors to consider.

Referee #1 (Remarks):
*I am satisfied by the responses, with one minor point:*
*The authors may want to consider that, although the nomenclature is not definitively established by any organization, it is most common among parabiosis investigators to use "isotypic" for mice of same genotype, and "heterotypic" for mice of different genotypes. It is most common to use "isochronic" for mice of same age joined together, regardless of genotype, and "heterochronic" for mice of different ages, regardless of genotype. So, given your experiments of mice of the same age but different genotypes joined in parabiosis, you might want to use the terms isochronic isotypic pairs and isochronic heterotypic pairs. (though many would simply drop the isochronic and just call them isotypic and heterotypic)*

Thank you for this suggestion. We agree and have changed the nomenclature of the parabiosis experiments to isotypic and heterotypic.

Referee #2 (Comments on Novelty/Model System):
*In this revision, the authors have conducted an important experiment demonstrating that circulating factors regulate enhanced metabolic rates in WAT and liver, which ultimately are responsible for decreased weight gain. Overall, the authors' findings are novel, the experiments are well done, and the manuscript is well written.*

We appreciate your thoughtful review and experimental suggestions to improve our manuscript.

Referee #3 (Remarks):
*The authors have done a great deal of additional work. While the primary question of mechanism remains, this is significant in potential impact. The parabiosis experiment is appreciatively difficult to perform and excludes some role of the nervous system in the observed phenotype. Future work will likely elucidate the mechanism by which the transgenic heart affects extra-cardiac tissues.*

Thank you for raising important issues in the first version of our manuscript. We appreciate the recommendations and think this has enhanced the quality of our final manuscript.
