# Supplementary material for: MED13-dependent signaling from the heart confers leanness by enhancing metabolism in adipose tissue and liver
Source: EMBO Mol Med. 2014 Nov 24;6(12):1610–21. doi: 10.15252/emmm.201404218 (PMC4287978; doi:10.15252/emmm.201404218)
Supplement: Supplementary file 3 — Source Data for Figure 2 B [file emmm0006-1610-sd3.pdf]

Supplementary figures for “MED13 dependent signaling from the heart confers leanness by enhancing metabolism in adipose tissue and liver.”

Content:

Figure S1. Cardiac overexpression of MED13 increases metabolic gene expression in white adipose tissue.

Figure S2. Regulation of liver metabolite production by cardiac expression of MED13 in the fed state.

Figure S3. Cardiac overexpression of MED13 alters metabolic gene expression.

Figure S4. MED13cTg hearts maintain the ability to adapt to fasting.

Figure S5. Circulating factor(s) regulate enhanced WAT and liver metabolism and contribute to the lean phenotype of MED13cTg mice.

Supplementary Table 1. Acylcarnitines in liver from fed and fasted WT and MED13cTg mice.

SupplementaryTable 2. Acyl-CoAs in liver from fed and fasted WT and MED13cTg mice.

SupplementaryTable 3. Acylcarnitines in heart from fed and fasted WT and MED13cTg mice.

Supplementary Table 4. Acyl-CoAs in heart from fed and fasted WT and MED13cTg mice.

SupplementaryTable 5. Acylcarnitines in serum from fed and fasted WT and MED13cTg mice.

Supplemental figure legends

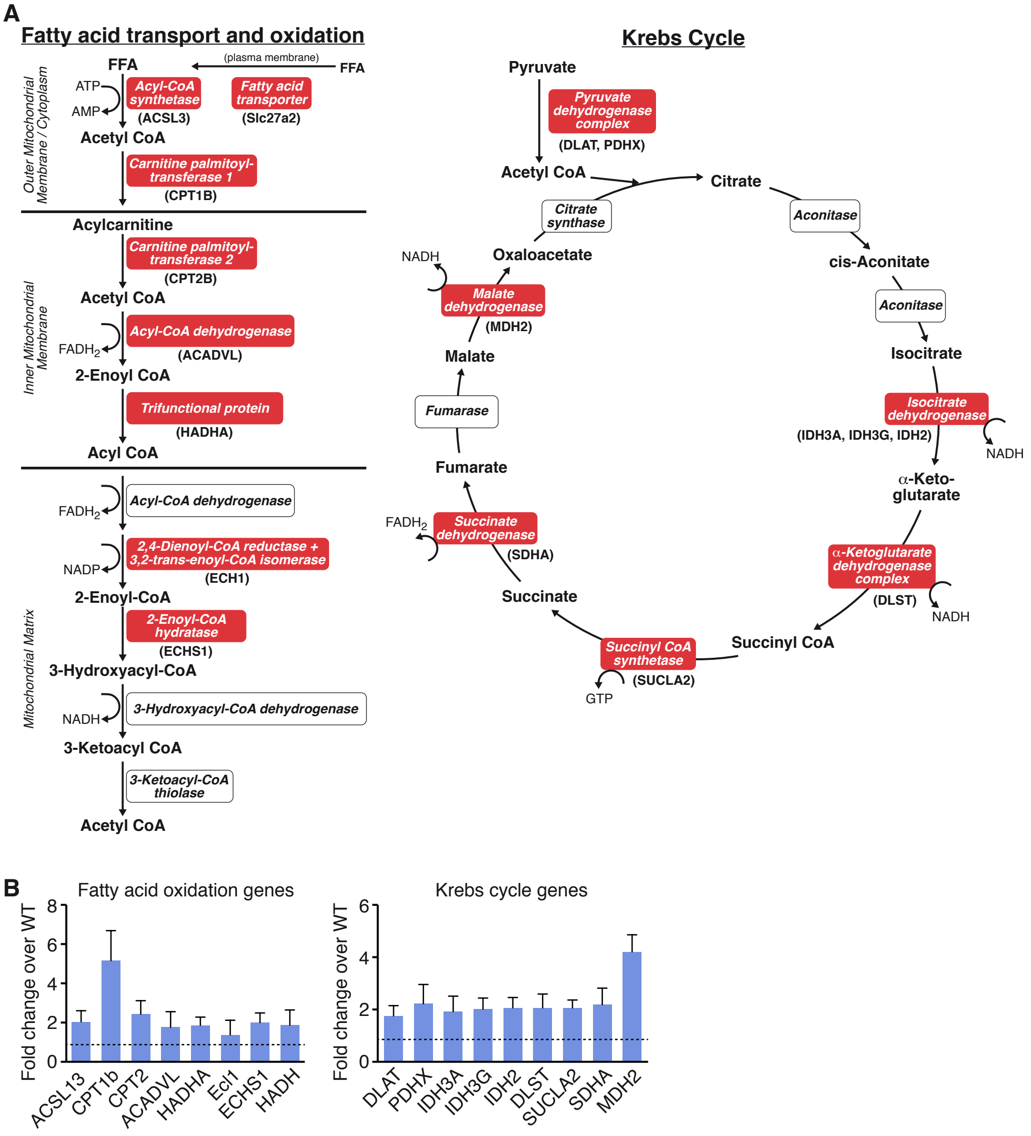
A
Fatty acid transport and oxidation
FFA
(plasma membrane)
FFA
Outer Mitochondrial Membrane / Cytoplasm
ATP
AMP
Acyl-CoA synthetase
(ACSL3)
Fatty acid transporter
(Slc27a2)
Acetyl CoA
Carnitine palmitoyl-transferase 1
(CPT1B)
Acylcarnitine
Inner Mitochondrial Membrane
Carnitine palmitoyl-transferase 2
(CPT2B)
Acetyl CoA
FADH2
Acyl-CoA dehydrogenase
(ACADVL)
2-Enoyl CoA
Trifunctional protein
(HADHA)
Acyl CoA
Mitochondrial Matrix
FADH2
Acyl-CoA dehydrogenase
NADP
2,4-Dienoyl-CoA reductase + 3,2-trans-enoyl-CoA isomerase
(ECH1)
2-Enoyl-CoA
2-Enoyl-CoA hydratase
(ECHS1)
3-Hydroxyacyl-CoA
NADH
3-Hydroxyacyl-CoA dehydrogenase
3-Ketoacyl CoA
3-Ketoacyl-CoA thiolase
Acetyl CoA
Krebs Cycle
Pyruvate
Pyruvate dehydrogenase complex
(DLAT, PDHX)
Acetyl CoA
Citrate
Citrate synthase
Aconitase
Oxaloacetate
NADH
Malate dehydrogenase
(MDH2)
cis-Aconitate
Aconitase
Malate
Isocitrate
Fumarase
Isocitrate dehydrogenase
(IDH3A, IDH3G, IDH2)
NADH
Fumarate
α-Keto-glutarate
FADH2
Succinate dehydrogenase
(SDHA)
α-Ketoglutarate dehydrogenase complex
(DLST)
NADH
Succinate
Succinyl CoA
Succinyl CoA synthetase
(SUCLA2)
GTP
B
Fatty acid oxidation genes
Fold change over WT
0
2
4
6
8
ACSL13
CPT1b
CPT2
ACADVL
HADHA
Ecl1
ECHS1
HADH
Krebs cycle genes
Fold change over WT
0
2
4
6
DLAT
PDHX
IDH3A
IDH3G
IDH2
DLST
SUCLA2
SDHA
MDH2

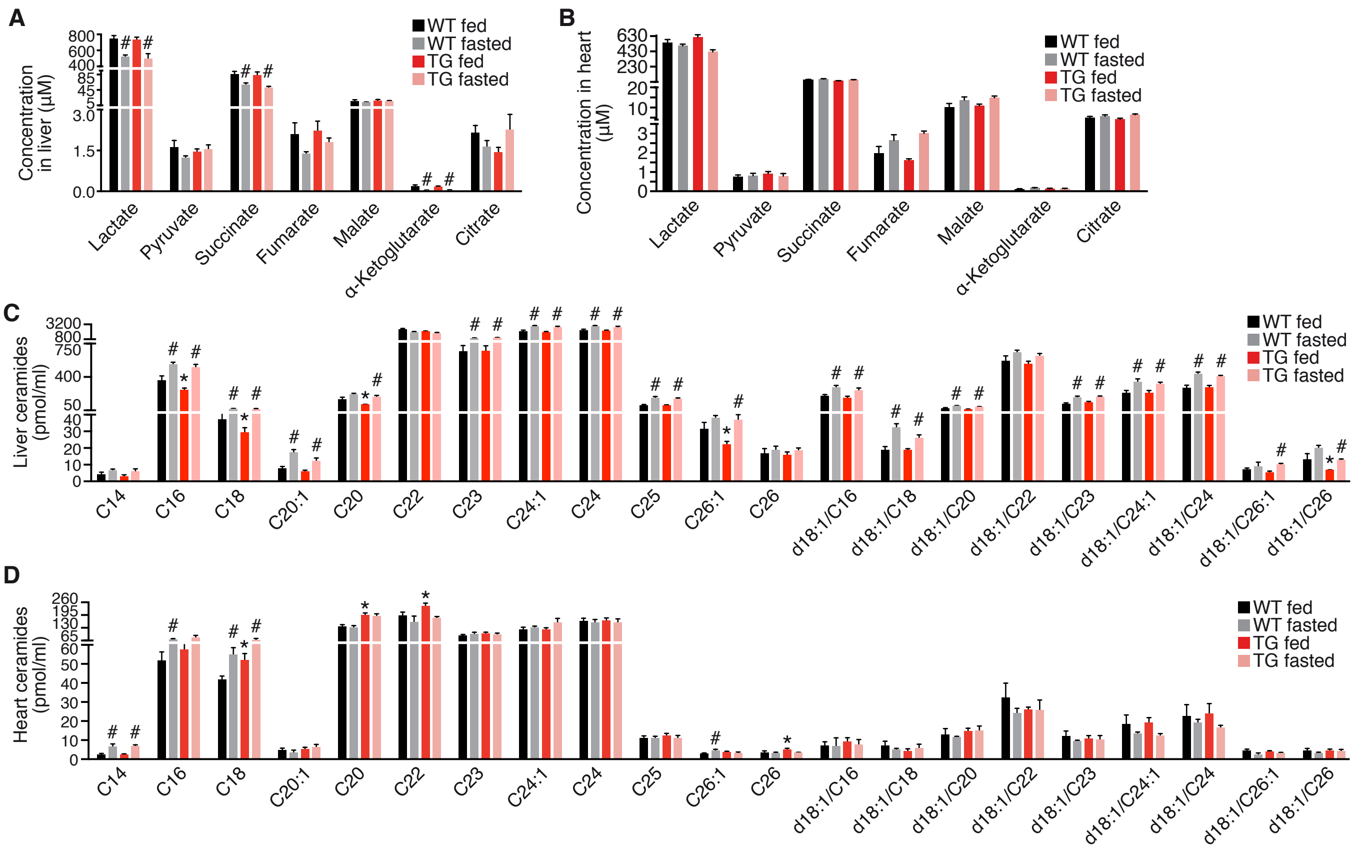
A
Concentration in liver (μM)
WT fed
WT fasted
TG fed
TG fasted
Lactate
Pyruvate
Succinate
Fumarate
Malate
α-Ketoglutarate
Citrate
B
Concentration in heart (μM)
C
Liver ceramides (pmol/ml)
C14
C16
C18
C20:1
C20
C22
C23
C24:1
C24
C25
C26:1
C26
d18:1/C16
d18:1/C18
d18:1/C20
d18:1/C22
d18:1/C23
d18:1/C24:1
d18:1/C24
d18:1/C26:1
d18:1/C26
D
Heart ceramides (pmol/ml)

SupplementaryFigure 3

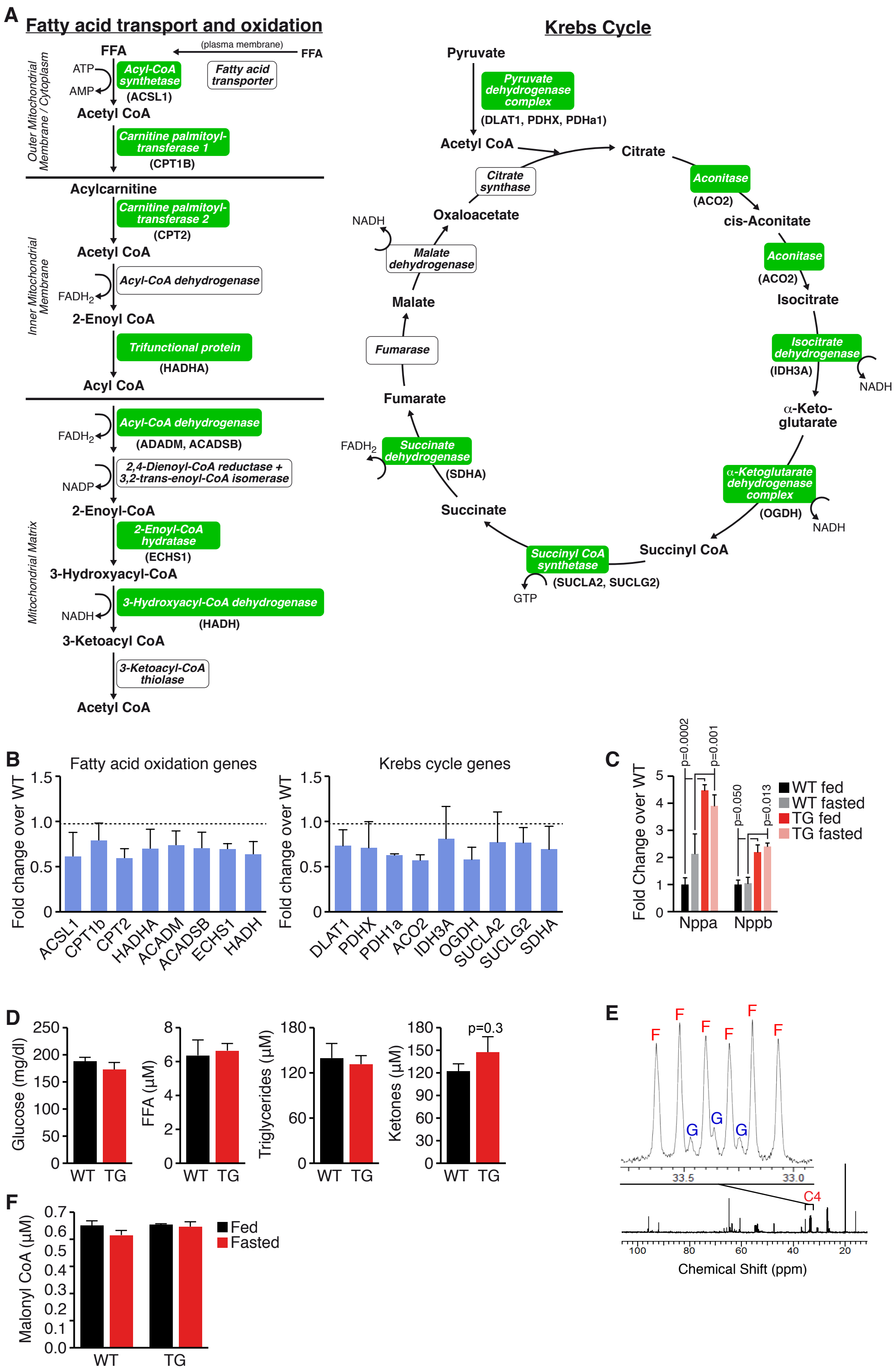

Supplementary Figure 4

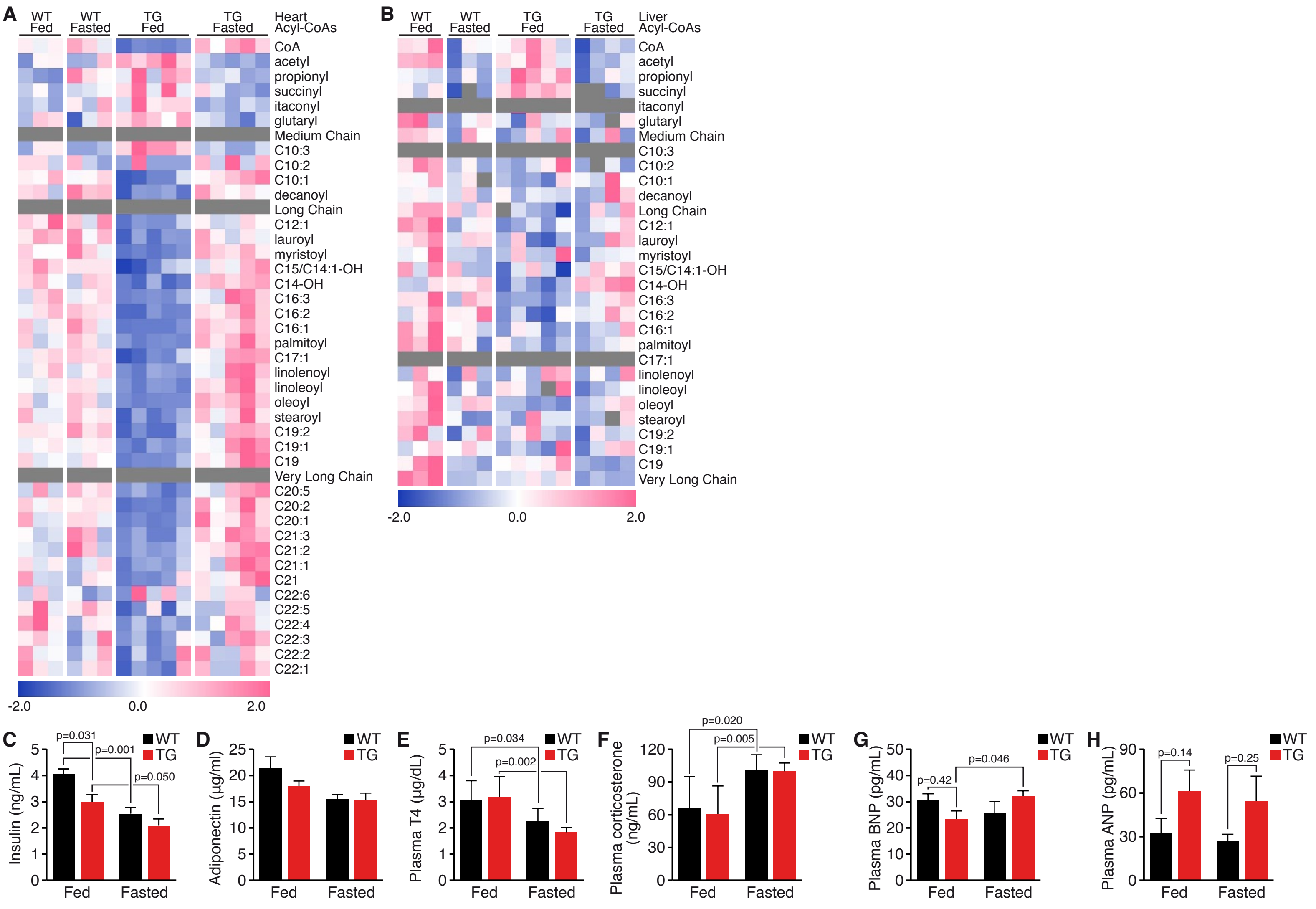

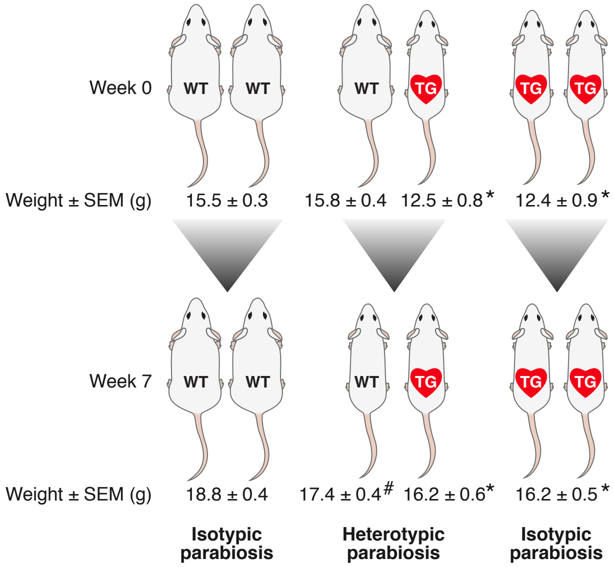
Week 0
WT
WT
WT
TG
TG
TG
Weight ± SEM (g)
15.5 ± 0.3
15.8 ± 0.4
12.5 ± 0.8*
12.4 ± 0.9*
Week 7
WT
WT
WT
TG
TG
TG
Weight ± SEM (g)
18.8 ± 0.4
17.4 ± 0.4#
16.2 ± 0.6*
16.2 ± 0.5*
Isotypic parabiosis
Heterotypic parabiosis
Isotypic parabiosis

Acylcarnitines in liver from fed and fasted WT and MED13cTg mice.

	WT Fed	WT Fasted	TG Fed	TG Fasted
C6	0.159 ± 0.020	0.025 ± 0.004 #	0.100 ± 0.020 *	0.022 ± 0.003 #
C5-OH/C3-DC	0.108 ± 0.010	0.071 ± 0.001 #	0.094 ± 0.013	0.067 ± 0.003 #
C4-DC/Ci4-DC	0.048 ± 0.003	0.027 ± 0.004 #	0.038 ± 0.004 *	0.030 ± 0.005
C8:1	0.003 ± 0.007	0.002 ± 0.000	0.003 ± 0.000	0.003 ± 0.000
C8	0.060 ± 0.004	0.041 ± 0.003 #	0.051 ± 0.005	0.032 ± 0.003 #
C6-DC/C8-OH	0.141 ± 0.010	0.207 ± 0.028 #	0.072 ± 0.011 *	0.237 ± 0.033 #
C10:3	0.005 ± 0.001	0.002 ± 0.004 #	0.003 ± 0.000	0.002 ± 0.000 #
C10	0.024 ± 0.003	0.016 ± 0.003	0.019 ± 0.003	0.017 ± 0.002
C8:1-DC	0.010 ± 0.001	0.008 ± 0.001	0.006 ± 0.001 *	0.013 ± 0.001 #
C10-OH/C8-DC	0.006 ± 0.001	0.006 ± 0.001	0.003 ± 0.001 *	0.009 ± 0.001 #
C12:1	0.005 ± 0.001	0.003 ± 0.001 #	0.002 ± 0.000 *	0.003 ± 0.000
C12	0.008 ± 0.001	0.008 ± 0.001	0.005 ± 0.001 *	0.008 ± 0.001 #
C14:1	0.015 ± 0.004	0.013 ± 0.003	0.006 ± 0.001 *	0.012 ± 0.001 #
C14	0.024 ± 0.004	0.026 ± 0.004	0.012 ± 0.002 *	0.024 ± 0.002 #
C16:2	0.008 ± 0.001	0.006 ± 0.001	0.005 ± 0.001 *	0.007 ± 0.001
C16:1	0.043 ± 0.010	0.049 ± 0.011	0.012 ± 0.001 *	0.040 ± 0.004 #
C16	0.106 ± 0.022	0.180 ± 0.017 #	0.050 ± 0.007 *	0.177 ± 0.013 #
C18:2	0.056 ± 0.012	0.086 ± 0.012	0.030 ± 0.004 *	0.109 ± 0.009 #
C18	0.069 ± 0.002	0.103 ± 0.005 #	0.026 ± 0.004 *	0.114 ± 0.003 #

Acyl-CoAs in liver from fed and fasted WT and MED13cTg mice.

	WT Fed	WT Fasted	TG Fed	TG Fasted
CoA	627.74 ± 49.08	560.66 ± 65.02	628.12 ± 43.57	461.86 ± 37.24 #
Acetyl	2865.00 ± 123.65	1272.68 ± 381.25 #	2395.40 ± 332.32	1065.10 ± 283.65 #
Propionyl	268.78 ± 19.40	241.51 ± 42.13	459.44 ± 64.43 *	234.23 ± 52.89 #
Crotonyl	59.52 ± 5.65	29.66 ± 12.56	89.31 ± 4.98 *	21.57 ± 13.04 #
C10:1	28.87 ± 5.73	17.25 ± 3.79	15.04 ± 3.29	10.15 ± 4.00
decanoyl	65.73 ± 6.83	55.31 ± 11.29	56.32 ± 10.48	50.21 ± 14.74
C12:1	38.22 ± 8.34	19.77 ± 4.71	29.04 ± 11.60	9.45 ± 5.15
lauroyl	52.58 ± 10.51	29.83 ± 10.75	24.94 ± 8.65 *	38.27 ± 3.72 #
myristoyl	52.69 ± 4.41	59.88 ± 8.03	53.31 ± 3.78	52.57 ± 10.89
C15/C14:1-OH	15.61 ± 2.11	19.64 ± 3.95	8.00 ± 2.54	15.15 ± 2.26
C16:1	166.55 ± 31.65	150.98 ± 28.32	104.16 ± 11.02	132.07 ± 17.03
palmitoyl	160.39 ± 14.14	160.57 ± 28.31	93.45 ± 10.74 *	155.28 ± 28.59
C17:1	28.28 ± 4.92	20.48 ± 2.67	26.66 ± 6.35	19.26 ± 2.70
linolenoyl	87.02 ± 8.52	85.43 ± 11.34	66.82 ± 8.95	86.57 ± 5.23
linoleoyl	530.14 ± 84.43 #	912.84 ± 121.60	450.37 ± 48.30 *	978.35 ± 60.50
oleoyl	707.33 ± 146.32	729.61 ± 161.64 #	294.78 ± 28.10 *	563.79 ± 78.89
stearoyl	181.68 ± 34.83	233.68 ± 27.71	126.34 ± 23.70	213.73 ± 25.01 #
C19:2	25.45 ± 5.93	23.04 ± 6.06	9.18 ± 2.86 *	16.40 ± 4.82
C19:1	44.09 ± 11.32	47.14 ± 9.29	34.69 ± 2.89	36.74 ± 2.74
C20:5	31.70 ± 2.49	27.02 ± 4.46	30.28 ± 3.70	29.05 ± 4.57
C20:2	201.48 ± 33.88	182.91 ± 69.01	157.05 ± 50.36	114.26 ± 21.42
C20:1	176.64 ± 57.69	207.02 ± 34.55	62.80 ± 10.10 *	142.40 ± 30.13 #
C21	24.66 ± 3.57	10.44 ± 4.04 #	14.90 ± 4.56	9.45 ± 4.43
C22:5	31.14 ± 5.68	37.36 ± 14.84	25.46 ± 5.23	19.39 ± 4.64
C22:4	37.38 ± 7.53	52.20 ± 4.80	41.87 ± 14.81	44.50 ± 10.89
C22:2	63.08 ± 14.78	35.18 ± 4.75	58.39 ± 6.61	25.89 ± 2.13 #
C22:1	147.35 ± 24.54	32.512 ± 4.89 #	67.85 ± 10.49 *	20.95 ± 5.65 #

Acylcarnitines in heart from fed and fasted WT and MED13cTg mice.

	WT Fed	WT Fasted	TG Fed	TG Fasted
C4/Ci4	0.602 ± 0.04	0.594 ± 0.03	0.334 ± 0.03 *	0.515 ± 0.07 #
C6	0.092 ± 0.01	0.098 ± 0.01	0.040 ± 0.01 *	0.097 ± 0.01 #
C8	0.031 ± 0.01	0.041 ± 0.01	0.007 ± 0.00 *	0.041 ± 0.00 #
C10	0.036 ± 0.01	0.077 ± 0.02	0.007 ± 0.00 *	0.070 ± 0.00 #
C10-OH/C8-DC	0.007 ± 0.00	0.013 ± 0.00 #	0.003 ± 0.00	0.011 ± 0.00 #
C12:1	0.021 ± 0.01	0.039 ± 0.01	0.007 ± 0.00 *	0.029 ± 0.00 #
C12	0.070 ± 0.03	0.140 ± 0.03	0.008 ± 0.00 *	0.119 ± 0.01 #
C12:1-OH/C10:1-DC	0.007 ± 0.00	0.012 ± 0.00	0.002 ± 0.00 *	0.012 ± 0.00 #
C12-OH/C10-DC	0.010 ± 0.00	0.014 ± 0.00	0.002 ± 0.00 *	0.019 ± 0.00 #
C14:1	0.136 ± 0.05	0.244 ± 0.06	0.016 ± 0.00 *	0.208 ± 0.01 #
C14	0.242 ± 0.10	0.504 ± 0.13	0.020 ± 0.01 *	0.523 ± 0.04 #
C14:2-OH/C12:2-DC	0.009 ± 0.00	0.014 ± 0.00	0.004 ± 0.00 *	0.014 ± 0.00 #
C14:1-OH/C12:1-DC	0.025 ± 0.01	0.049 ± 0.01	0.005 ± 0.00 *	0.054 ± 0.01 #
C14-OH/C12-DC	0.023 ± 0.01	0.036 ± 0.01	0.003 ± 0.00 *	0.050 ± 0.00 #
C16:2	0.191 ± 0.05	0.315 ± 0.06	0.016 ± 0.00 *	0.333 ± 0.05 #
C16:1	0.380 ± 0.16	0.781 ± 0.20	0.020 ± 0.01 *	0.816 ± 0.09 #
C16	0.674 ± 0.31	1.821 ± 0.54	0.051 ± 0.01 *	2.368 ± 0.33 #
C16-OH/C14-DC	0.054 ± 0.02	0.106 ± 0.03	0.007 ± 0.00 *	0.193 ± 0.02 #
C18:3	0.056 ± 0.02	0.069 ± 0.01	0.006 ± 0.00 *	0.096 ± 0.02 #
C18:2	0.707 ± 0.20	1.402 ± 0.36	0.051 ± 0.01 *	1.989 ± 0.40 #
C18:1	0.889 ± 0.37	2.163 ± 0.61	0.016 ± 0.01 *	2.805 ± 0.45 #
C18	0.288 ± 0.12	0.609 ± 0.17	0.023 ± 0.01 *	0.784 ± 0.11 #
C20:2	0.254 ± 0.09	0.479 ± 0.13	0.018 ± 0.01 *	0.598 ± 0.12 #
C20:1	0.227 ± 0.11	0.466 ± 0.13	0.015 ± 0.01 *	0.560 ± 0.09 #

Acyl-CoAs in heart from fed and fasted WT and MED13cTg mice.

	WT Fed	WT Fasted	TG Fed	TG Fasted
CoA	271.06 ± 34.34	338.18 ± 30.21	179.54 ± 6.56 *	390.04 ± 27.67 #
Acetyl	474.45 ± 93.18	327.22 ± 101.0	687.53 ± 63.0 *	275.97 ± 27.43 #
Propionyl	17.12 ± 4.05	23.98 ± 4.51	27.43 ± 3.93 *	16.69 ± 0.98 #
Succinyl	57.54 ± 4.64	47.79 ± 8.28	79.84 ± 11.50 *	51.42 ± 6.27 #
Itaconyl	4.02 ± 1.76	4.18 ± 1.44	6.52 ± 1.78	1.89 ± 0.43 #
glutaryl	15.95 ± 1.91	11.12 ± 2.52	16.75 ± 0.98	11.21 ± 0.92 #
C10:3	5.62 ± 1.14	1.88 ± 0.46 #	9.52 ± 1.07 *	3.06 ± 0.55 #
C10:2	3.54 ± 0.53	1.64 ± 1.10	1.89 ± 1.32	4.16 ± 1.18
C10:1	15.81 ± 3.56	18.28 ± 1.34	7.13 ± 2.21 *	24.25 ± 2.43 #
decanoyl	38.34 ± 7.07	59.49 ± 8.01	19.95 ± 3.06 *	52.67 ± 4.85 #
C12:1	28.42 ± 7.82	28.52 ± 5.38	16.06 ± 1.63 *	26.41 ± 1.90 #
lauroyl	52.05 ± 13.09	63.07 ± 11.47	27.42 ± 3.06 *	59.01 ± 6.34 #
myristoyl	76.07 ± 18.69	120.33 ± 18.84	32.77 ± 2.94 *	124.83 ± 8.66 #
C15/C14:1-OH	14.86 ± 3.170	15.98 ± 0.23	9.37 ± 1.47 *	16.51 ± 1.57 #
C14-OH	8.60 ± 1.91	9.35 ± 1.13	3.90 ± 0.49 *	12.45 ± 0.55 #
C16:3	21.51 ± 5.62	19.03 ± 2.38	6.83 ± 1.06 *	27.78 ± 5.14 #
C16:2	152.37 ± 37.62	190.59 ± 13.47	61.55 ± 6.90 *	216.98 ± 19.08 #
C16:1	233.90 ± 67.36	372.93 ± 58.76	69.14 ± 8.50 *	412.00 ± 28.50 #
palmitoyl	267.85 ± 71.30	435.52 ± 57.45	101.60 ± 9.31 *	570.67 ± 59.63 #
C17:1	27.310 ± 5.17	32.37 ±2.87	18.19 ± 2.24 *	37.65 ± 3.24 #
linolenoyl	128.16 ± 28.28	124.50 ± 15.86	44.19 ± 6.03 *	185.20 ± 33.51 #
linoleoyl	885.23 ± 173.5	1220.09 ± 91.81	347.38 ± 42.00 *	1725.87 ± 234.11 #
oleoyl	865.37 ± 239.0	1456.93 ± 227.0	285.47 ± 19.37 *	1999.52 ± 159.78 #
stearoyl	217.20 ± 44.62	303.69 ± 18.74	133.51 ± 15.98 *	323.37 ± 37.25 #
C19:2	16.93 ± 3.53	23.06 ± 3.88	6.91 ± 1.69 *	23.84 ± 3.07 #
C19:1	106.40 ± 23.76	126.38 ± 6.23	55.12 ± 6.52 *	187.37 ± 27.20 #
C19	79.82 ± 18.68	97.96 ± 7.88	37.39 ± 6.46 *	149.15 ± 21.28 #
C20:5	5.18 ± 1.05	6.47 ± 0.70	1.76 ± 0.47 *	7.17 ± 1.19 #
C20:2	354.75 ± 84.42	465.09 ± 23.43	124.58 ± 23.24 *	581.61 ± 75.80 #
C20:1	229.03 ± 68.53	331.23 ± 29.15	84.71 ± 13.94 *	409.89 ± 54.48 #
C21:3	9.00 ± 1.22	17.42 ± 4.58	3.11 ± 1.44 *	21.15 ± 2.60 #
C21:2	7.53 ± 1.99	17.34 ± 5.52	3.13 ± 1.22 *	21.60 ± 3.05 #
C21:1	36.85 ± 8.74	40.91 ± 5.20	24.07 ± 3.00 *	62.33 ± 10.22 #
C21	31.22 ± 7.42	32.22 ± 4.29	19.24 ± 5.61 *	46.84 ± 6.73 #
C22:6	7.25 ± 1.78	6.27 ± 1.05	10.99 ± 3.42	9.71 ± 1.46
C22:5	4.07 ± 1.48	5.88 ± 0.53	2.43 ± 0.76	4.55 ± 0.69
C22:4	13.68 ± 4.19	12.03 ± 2.00	5.63 ± 0.82 *	14.01 ± 2.62 #
C22:3	20.17 ± 3.28	21.18 ± 5.39	13.58 ± 2.22 *	25.74 ± 4.25 #
C22:2	56.64 ± 14.63	65.15 ± 12.36	45.61 ± 16.42	70.02 ± 11.65
C22:1	43.81 ± 8.16	48.97 ± 5.10	30.97 ± 7.82	48.91 ± 6.24

Acylcarnitines in serum from fed and fasted WT and MED13cTg mice.

	**WT Fed**	**WT Fasted**	**TG Fed**	**TG Fasted**
C2	23.450 ± 2.80	43.736 ± 4.55 #	21.592 ± 1.98	42.331 ± 2.26 #
C3	1.320 ± 0.140	0.623 ± 0.049 #	1.446 ± 0.119	0.628 ± 0.056 #
C4/Ci4	1.670 ± 0.091	1.079 ± 0.108 #	1.225 ± 0.155	0.939 ± 0.116
C5:1	0.240 ± 0.025	0.039 ± 0.004 #	0.224 ± 0.050	0.042 ± 0.005 #
C5	0.164 ± 0.010	0.168 ± 0.023	0.233 ± 0.021	0.188 ± 0.012
C4-OH	0.174 ± 0.019	0.476 ± 0.057 #	0.207 ± 0.042	0.489 ± 0.050 #
C6	0.164 ± 0.019	0.199 ± 0.026	0.115 ± 0.015	0.179 ± 0.026
C5-OH/C3-DC	0.092 ± 0.015	0.169 ± 0.012 #	0.121 ± 0.017	0.180 ± 0.017 #
C4-DC/Ci4-DC	0.053 ± 0.006	0.082 ± 0.011 #	0.065 ± 0.010	0.068 ± 0.014
C8:1	0.023 ± 0.002	0.009 ± 0.002 #	0.020 ± 0.003	0.012 ± 0.002 #
C8	0.148 ± 0.038	0.035 ± 0.003 #	0.162 ± 0.049	0.035 ± 0.006 #
C10-OH/C8-DC	0.156 ± 0.022	0.020 ± 0.002 #	0.151 ± 0.049	0.023 ± 0.003 #
C12:1	0.037 ± 0.006	0.045 ± 0.007	0.033 ± 0.009	0.048 ± 0.007
C12:2-OH/C10:2-DC	0.019 ± 0.002	0.040 ± 0.005 #	0.014 ± 0.002	0.039 ± 0.005 #
C12:1-OH/C10:1-DC	0.034 ± 0.009	0.132 ± 0.021 #	0.032 ± 0.009	0.120 ± 0.014 #
C12-OH/C10-DC	0.067 ± 0.008	0.134 ± 0.022 #	0.060 ± 0.008	0.136 ± 0.014 #
C14	0.050 ± 0.010	0.168 ± 0.029 #	0.038 ± 0.013	0.151 ± 0.015 #
C14:3-OH/C12:3-DC	0.240 ± 0.028	0.437 ± 0.042 #	0.248 ± 0.018	0.427 ± 0.039 #
C14:2-OH/C12:2-DC	0.008 ± 0.002	0.025 ± 0.004 #	0.010 ± 0.002	0.027 ± 0.003 #
C14-OH/C12-DC	0.065 ± 0.012	0.172 ± 0.016 #	0.071 ± 0.009	0.194 ± 0.016 #
C16:3	0.094 ± 0.019	0.386 ± 0.033 #	0.098 ± 0.026	0.404 ± 0.029 #
C16:2-OH/C14:2-DC	0.028 ± 0.006	0.037 ± 0.003 #	0.022 ± 0.006	0.038 ± 0.003 #

SUPPLEMENTARY FIGURE LEGENDS

Figure S1. Cardiac overexpression of MED13 increases metabolic gene expression in white adipose tissue. (A) Schematic of changes in expression of genes involved in fatty acid transport and oxidation and the Krebs cycle. Red indicates increased gene expression in MED13cTg WAT, gene abbreviations are in parentheses and shown in graphical form in Figure 1. (B) Gene expression was also validated by qPCR, n=6. Data are mean + SEM and all genes shown in the graphs are significantly increased in MED13cTg WAT ($p<0.05$), *t*-test. Related to Figure 2.

Figure S2. Regulation of liver metabolite production by cardiac expression of MED13 in the fed state. (A) Krebs cycle intermediary metabolites are unchanged in the fed state and are decreased in both WT and MED13cTg liver with fasting, but (B) are unchanged in the heart. (C) Ceramide species in the liver and (D) hearts of fed and fasted mice, n=5. Data are mean + SEM, two-way ANOVA followed by Tukey's test, *$p<0.05$ TG fed vs. WT fed, #$p<0.05$ fed vs. fasted. Related to Figure 3.

Figure S3. Cardiac overexpression of MED13 alters metabolic gene expression. (A) Schematic of changes in expression of genes involved in fatty acid oxidation and the Krebs cycle. Green indicates decreased gene expression in MED13cTg heart, gene abbreviations are in parentheses and shown in graphical form in Figure 4. (B) Fatty acid oxidation and the Krebs cycle gene expression was validated by qPCR. All genes shown in the graphs are significantly decreased in MED13cTg heart ($p<0.05$), t-test. (C) Nppa and Nppb gene expression trended to increase in MED13cTg hearts, irrespective of the fed or fasted state, n=5. (D) Blood glucose, free fatty acids (FFA), triglycerides, and ketones in the postprandial state, n=6. (E) Representative 13C-NMR spectrum of a perfused heart. The enlarged portion of the spectra shows the C4 glutamate peaks that arise from fatty acid oxidation (indicated by F) and glucose oxidation (indicated by G). (F) Malonyl CoA levels in WT and Med13cTg hearts in the fed and fasted state. Data are mean + SEM, t-test or two-way ANOVA followed by Dunnett's test. Related to Figure 4.

Figure S4. MED13cTg hearts maintain the ability to adapt to fasting. (A) Acyl-CoA species in heart and (B) liver are decreased with fasting in MED13cTg mice. (C) Insulin, (D) adiponectin, (E) thyroxine (T4), (F) corticosterone, (G) brain natriuretic peptide (BNP), and (H) atrial natriuretic peptide (ANP) in serum from fed and fasted mice, n=5. Data are mean + SEM, two-way ANOVA followed by Tukey's test, *$p<0.05$ TG fed vs. WT fed, #$p<0.05$ fed vs. fasted. Related to Figure 5.

Figure S5. Circulating factor(s) regulate enhanced WAT and liver metabolism and contribute to the lean phenotype of MED13cTg mice. Schematic and results of isotypic and heterotypic parabiosis experiments in WT and MED13cTg mice. Weights are from individual parabiots before surgery and 7 weeks post-surgery, n=6 pair per group. Data are mean ± SEM,

two-way ANOVA followed by Dunnett's test, *p<0.05 TG vs. WT, #p<0.05 heterotypic WT parabiots vs. isotypic WT parabiots. Related to Figure 6.

Supplementary Table 1. Acylcarnitines in liver from fed and fasted WT and MED13cTg mice. Acylcarnitines (μM) measured by metabolomics are mean ± SEM, two-way ANOVA followed by Tukey's test, n=5, *p<0.05 TG fed vs. WT fed, #p<0.05 fed vs. fasted.

Supplementary Table 2. Acyl-CoAs in liver from fed and fasted WT and MED13cTg mice. Acyl-CoAs (pmol/ml) measured by metabolomics are mean ± SEM, two-way ANOVA followed by Tukey's test, n=5, *p<0.05 TG fed vs. WT fed, #p<0.05 fed vs. fasted.

Supplementary Table 3. Acylcarnitines in heart from fed and fasted WT and MED13cTg mice. Acylcarnitines (μM) measured by metabolomics are mean ± SEM, two-way ANOVA followed by Tukey's test, n=5, *p<0.05 TG fed vs. WT fed, #p<0.05 fed vs. fasted.

Supplementary Table 4. Acyl-CoAs in heart from fed and fasted WT and MED13cTg mice. Acyl-CoAs (pmol/ml) measured by metabolomics are mean ± SEM, two-way ANOVA followed by Tukey's test, n=5, *p<0.05 TG fed vs. WT fed, #p<0.05 fed vs. fasted.

SupplementaryTable 5. Acylcarnitines in serum from fed and fasted WT and MED13cTg mice. Acylcarnitines (μM) measured by metabolomics are mean ± SEM, two-way ANOVA followed by Tukey's test, n=5, *p<0.05 TG fed vs. WT fed, #p<0.05 fed vs. fasted.
