# Supplementary material for: MED13-dependent signaling from the heart confers leanness by enhancing metabolism in adipose tissue and liver
Source: EMBO Mol Med. 2014 Nov 24;6(12):1610–21. doi: 10.15252/emmm.201404218 (PMC4287978; doi:10.15252/emmm.201404218)
Supplement: Supplementary file 2 — Review Process File [file emmm0006-1610-sd2.pdf]

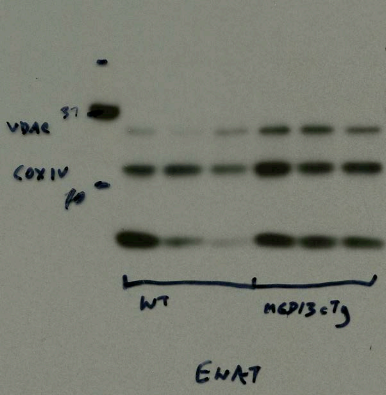

37
VDAC
COX IV
10
WT
MCD13cTg
ENAT
